# Supplementary material for: The Cellular Microbiome of Visceral Organs: An Inherent Inhabitant of Parenchymal Cells
Source: Microorganisms. 2024 Jun 29;12(7):1333. doi: 10.3390/microorganisms12071333 (PMC11279389; doi:10.3390/microorganisms12071333)
Supplement: Supplementary file 1 [file microorganisms-12-01333-s001.zip › supplementary figures.pdf]

Supplementary figures

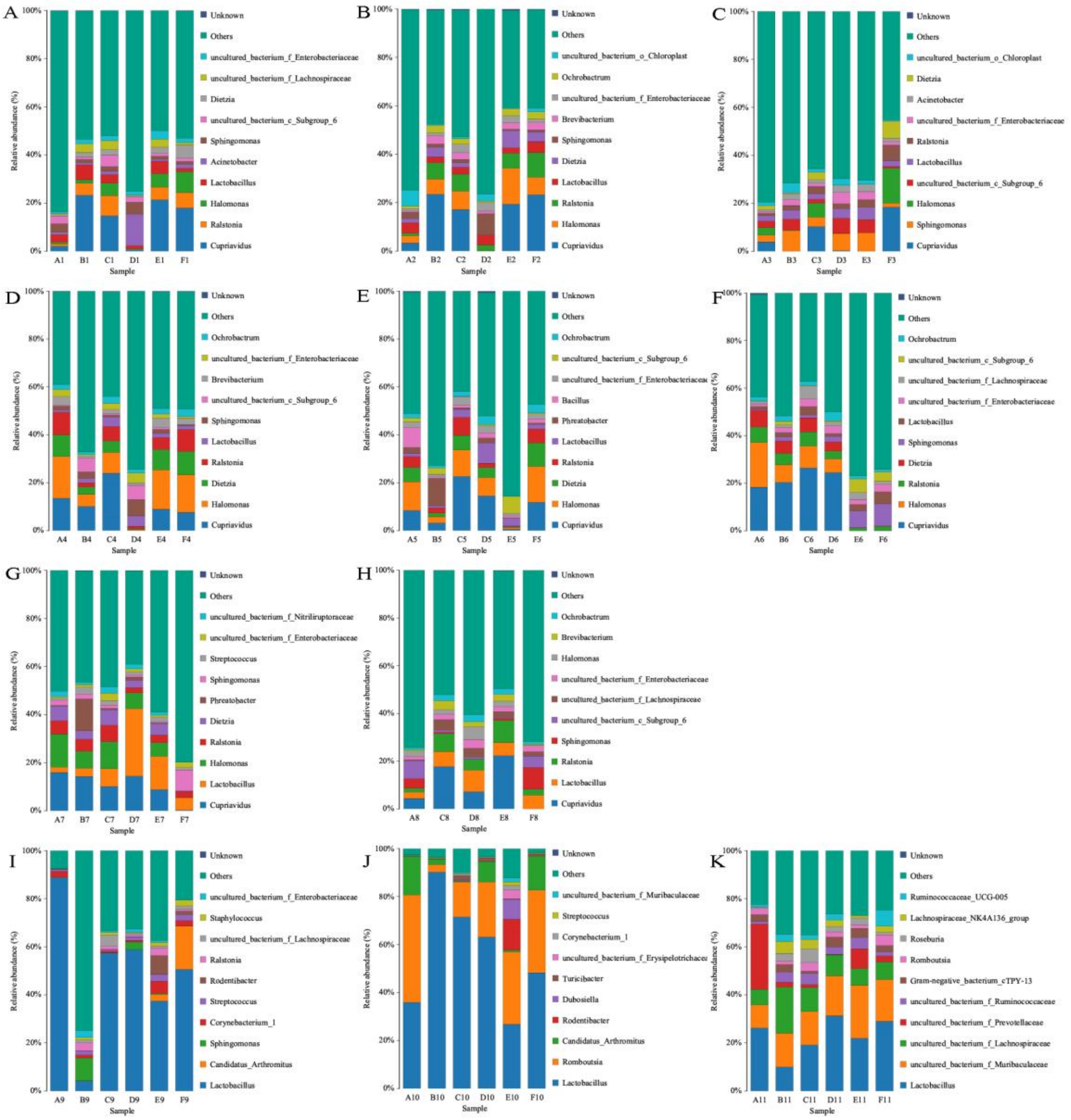

Figure S1. Variation of visceral bacteria across individuals (genera, n = 6). A, brain; B, heart; C, liver; D, spleen; E, lung; F, kidney; G, pancreas; H, skeletal muscle; I, jejunum contents; J, ileum contents; and K, feces. Horizontal axes represent six rats.

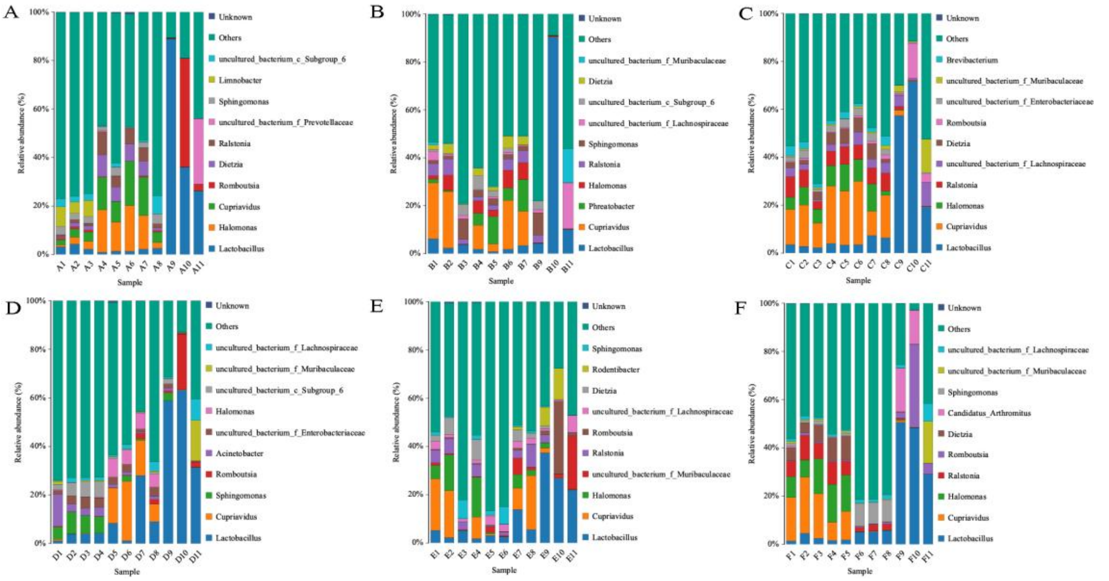

Figure S2. Variation of visceral bacteria in an individual across organs (genera). A to F represent six rats. In each rat: 1, brain; 2, heart; 3, liver; 4, spleen; 5, lung; 6, kidney; 7, pancreas; 8, skeletal muscle; 9, jejunum contents; 10, ileum contents; and 11, feces. Horizontal axes represent visceral organs.

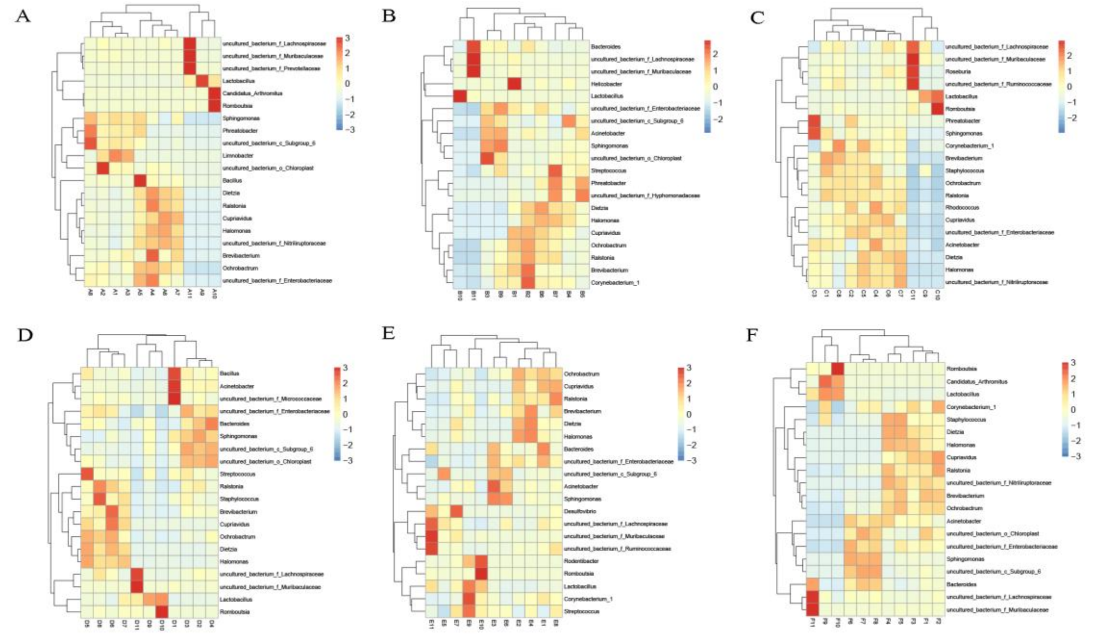

Figure S3. Relationship of visceral bacteria in an individual across organs (genera). A to F represent six rats. In each rat: 1, brain; 2, heart; 3, liver; 4, spleen; 5, lung; 6, kidney; 7, pancreas; 8, skeletal muscle; 9, jejunum contents; 10, ileum contents; and 11, feces.

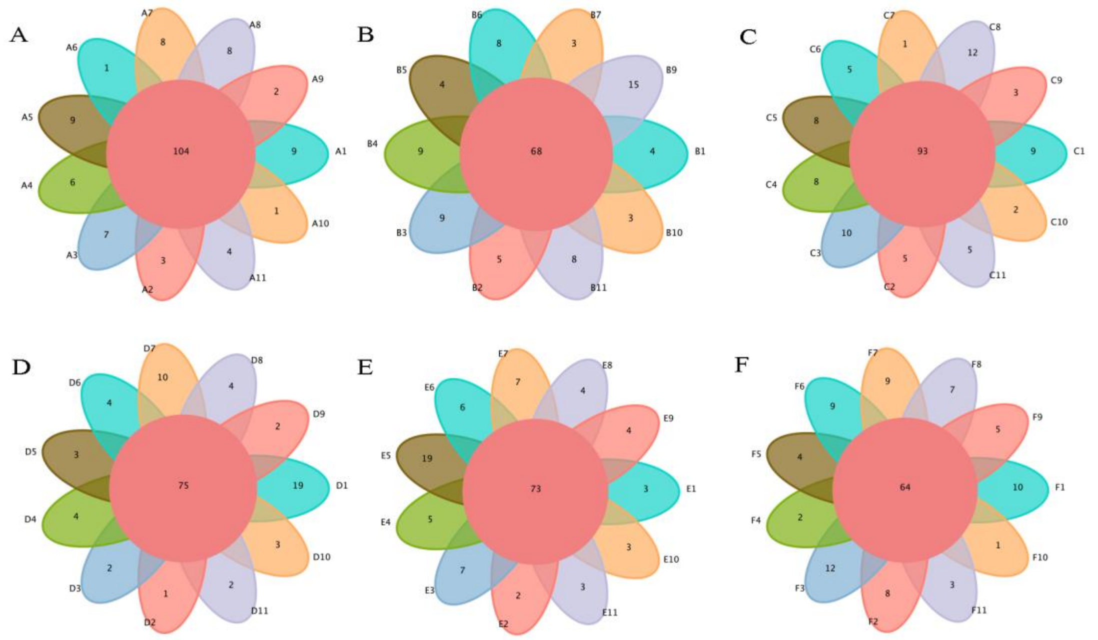

Figure S4. The prevalence of visceral bacteria in an individual across organs (genera). Each organ contained small numbers of exclusive bacteria with a low abundance compared to other organs. A to F represent six rats. In each rat: 1, brain; 2, heart; 3, liver; 4, spleen; 5, lung; 6, kidney; 7, pancreas; 8, skeletal muscle; 9, jejunum; 10, ileum; and 11, colon.

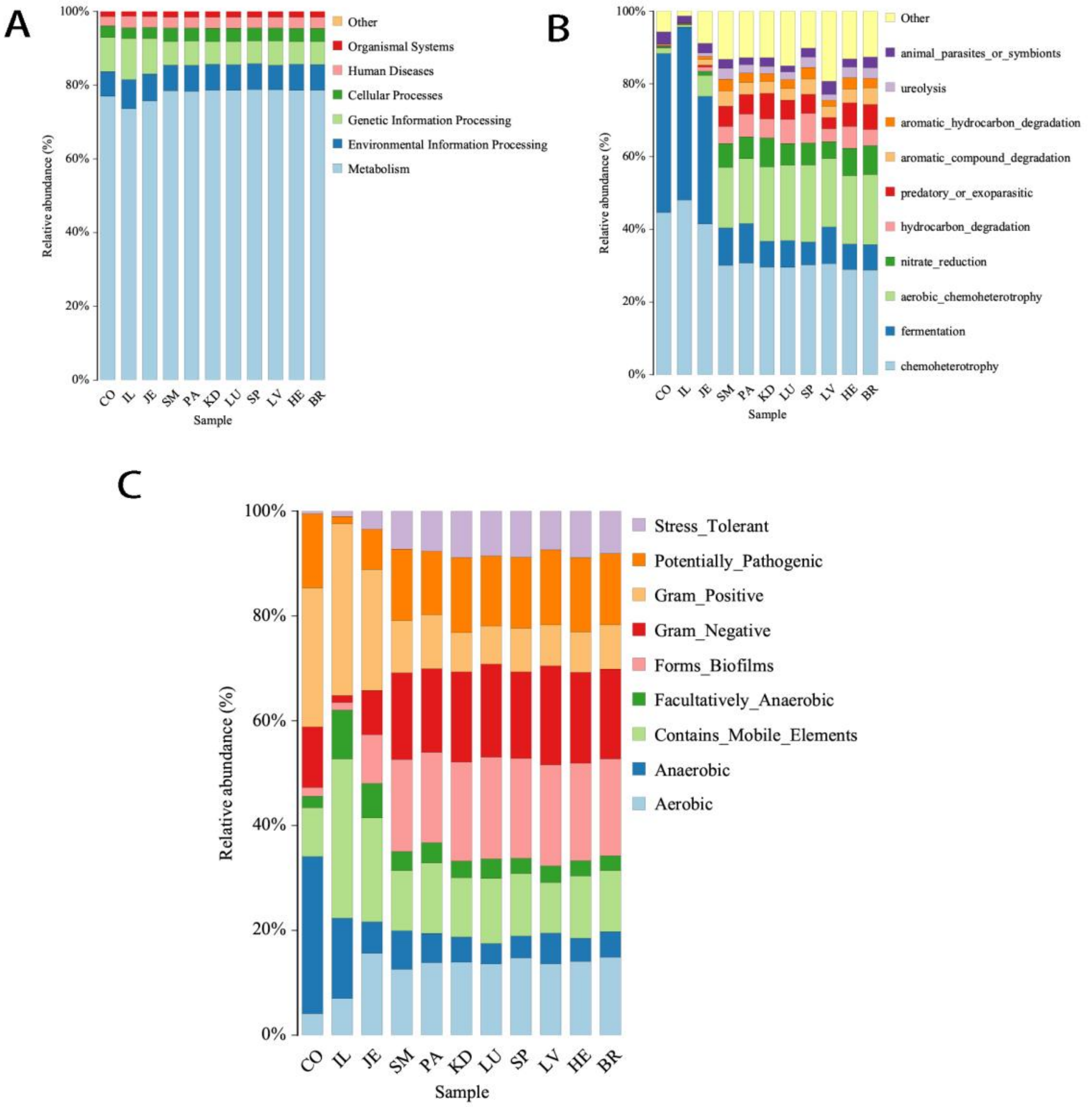

Figure S5. Genome functional composition of visceral bacteria of adult SD rats. The visceral bacteria showed completely different genome functional features from those of intestinal microbiota. (A) Picrust2; (B) FARPROTAX; and (C) Bugbase.

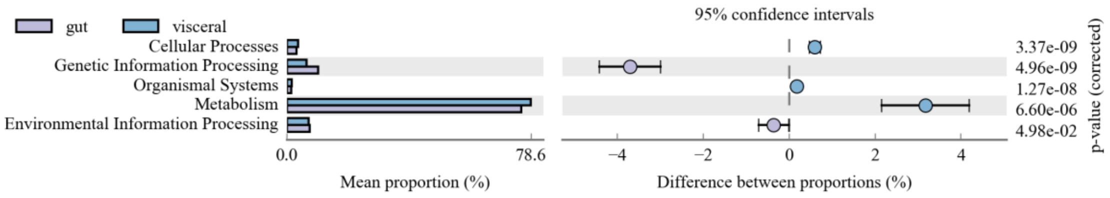

Figure S6. Picrust2 comparison of visceral and gut microbiota. The genome functional composition of cellular processes, organ systems, and metabolism of visceral bacteria were higher than those of the gut microbiota.

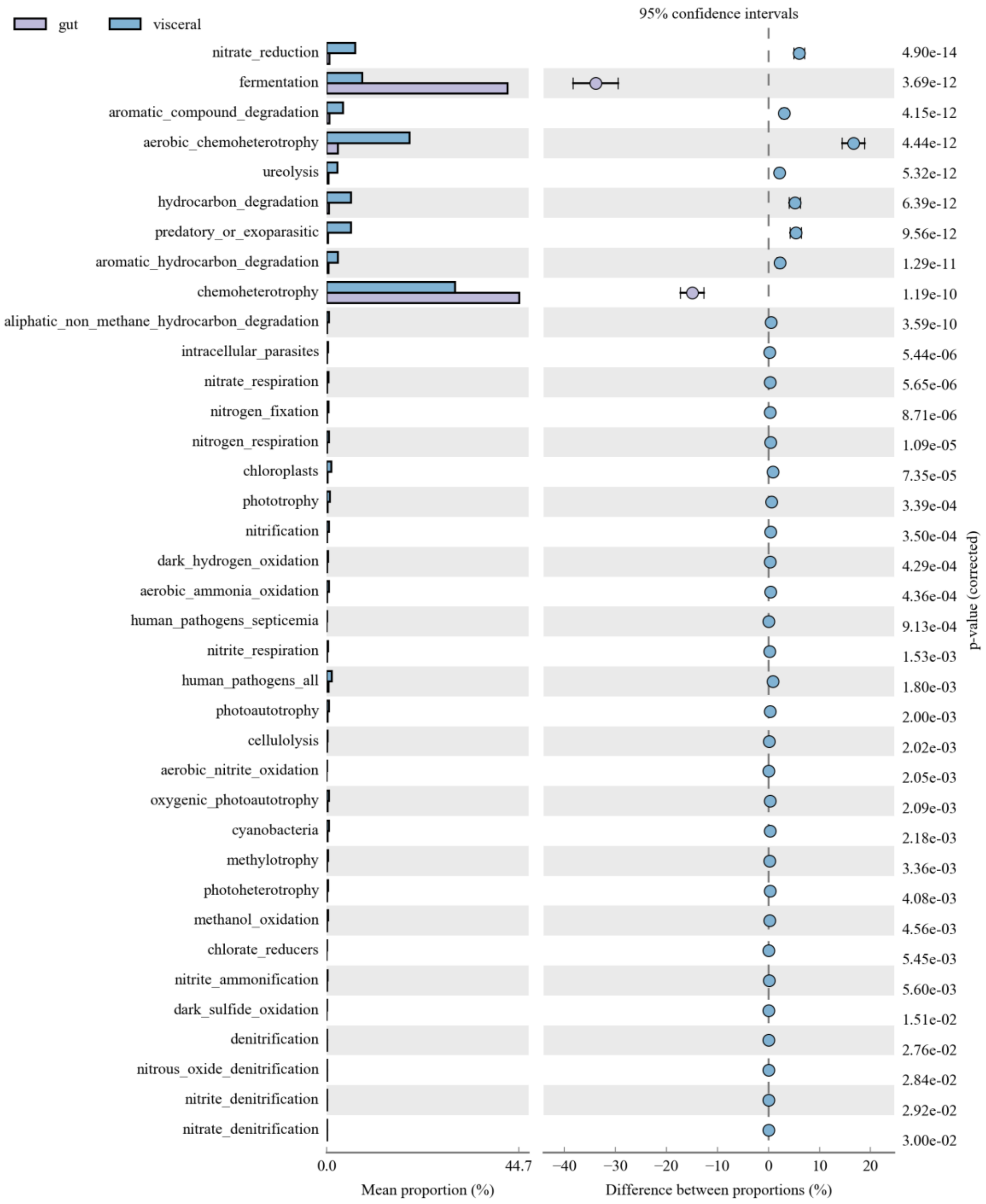

Figure S7. FARPROTAX analysis between visceral and gut microbiota genome functional composition. Visceral "fermentation" and "chemoheterotrophy" were lower than in intestinal bacteria, and "aerobic chemoheterotrophy", "nitrate reduction", "hydrocarbon degradation", "predatory or exoparasitic", "aromatic compound degradation", "aromatic hydrocarbon degradation", and "ureolysis" were higher than in intestinal bacteria.

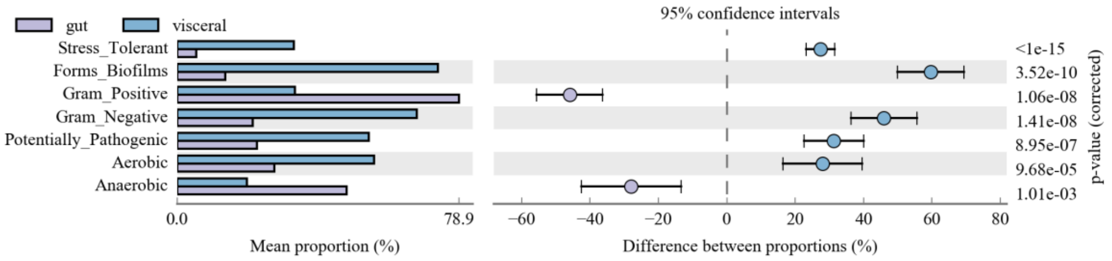

Figure S8. Genome functional composition of visceral bacteria of adult SD rats (Bugbase analysis). The intestinal bacteria showed a high abundance of "anaerobic", "gram positive", "facultatively anaerobic" (jejunum and ileum), and "contains mobile elements" (jejunum and ileum), while the visceral bacteria were abundant in "potentially pathogenic", "forms biofilms", "gram negative", and "aerobic and stress tolerant."

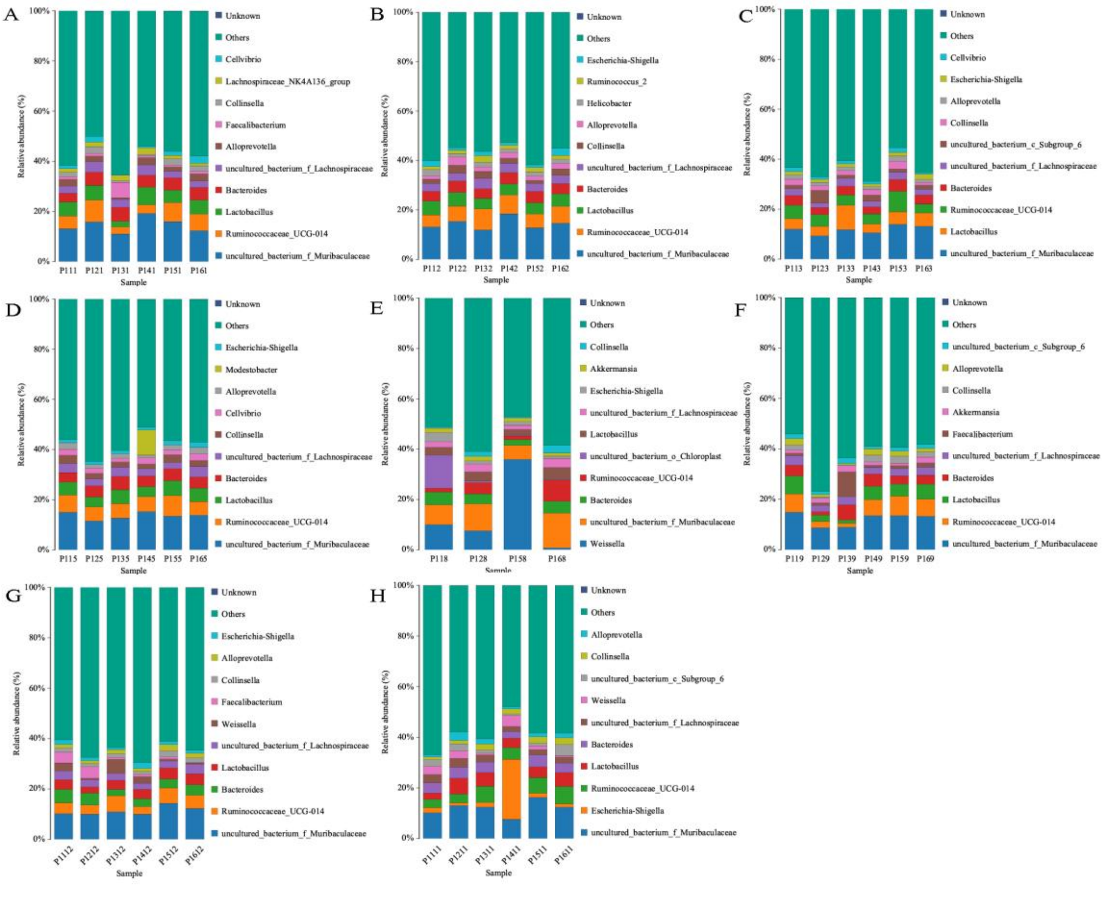

Figure S9. Variation of visceral organ bacteria across individuals in the same litter (genera, n = 6). The visceral bacteria in specific organs were constant across individuals, except for the skeletal muscles and milk clot. A, brain; B, HSKP (heart, spleen, kidney, pancreas); C, liver; D, lung; E, skeletal muscle (P128 was combination of three samples P128, P138 and P148); F, intestine tissue; G, skin tissue; and H, milk clot. Sample P128 in E was a mixture from three individuals. Horizontal axes represent six newborn rats.

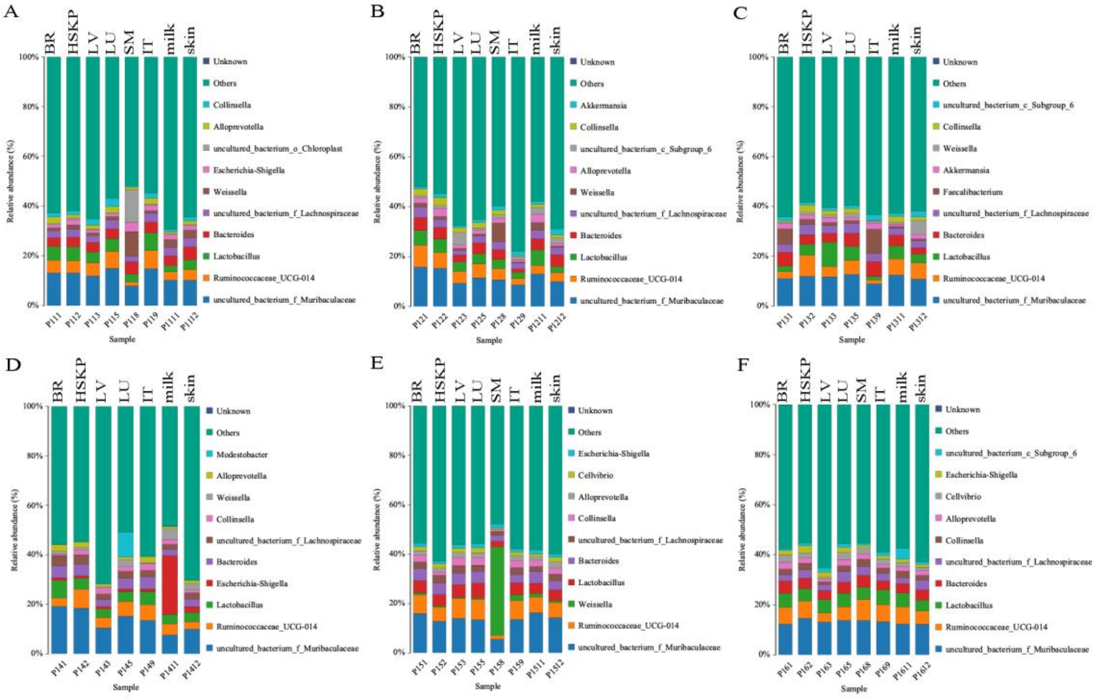

Figure S10. Visceral bacteria of newborn rats across organs (genera). A to F represent six newborn rats from the same litter. There was litter variation across organs in each individual except the milk in rat D and skeletal muscle in rat E. Skeletal muscle of rat B is the mixture of rat B, C, and D due to the sample amount demanding. Horizontal axes represent visceral organs, skin tissue and milk clot.

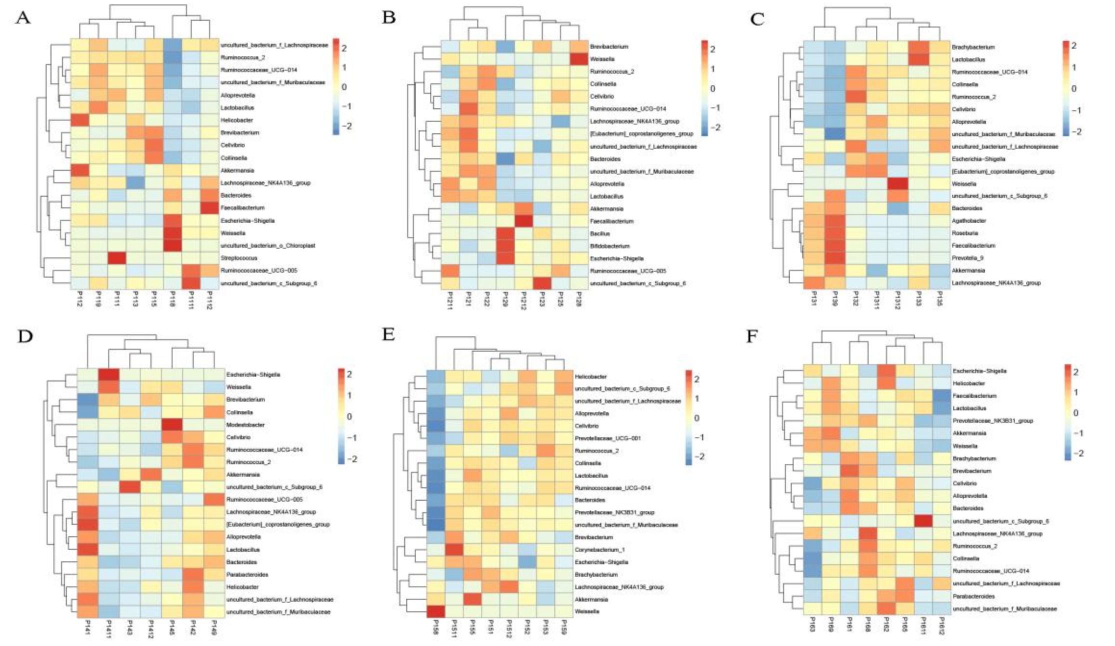

Figure S11. Relationship of newborn rat visceral bacteria across organs (genera). A to F represent six newborn rats from the same litter.

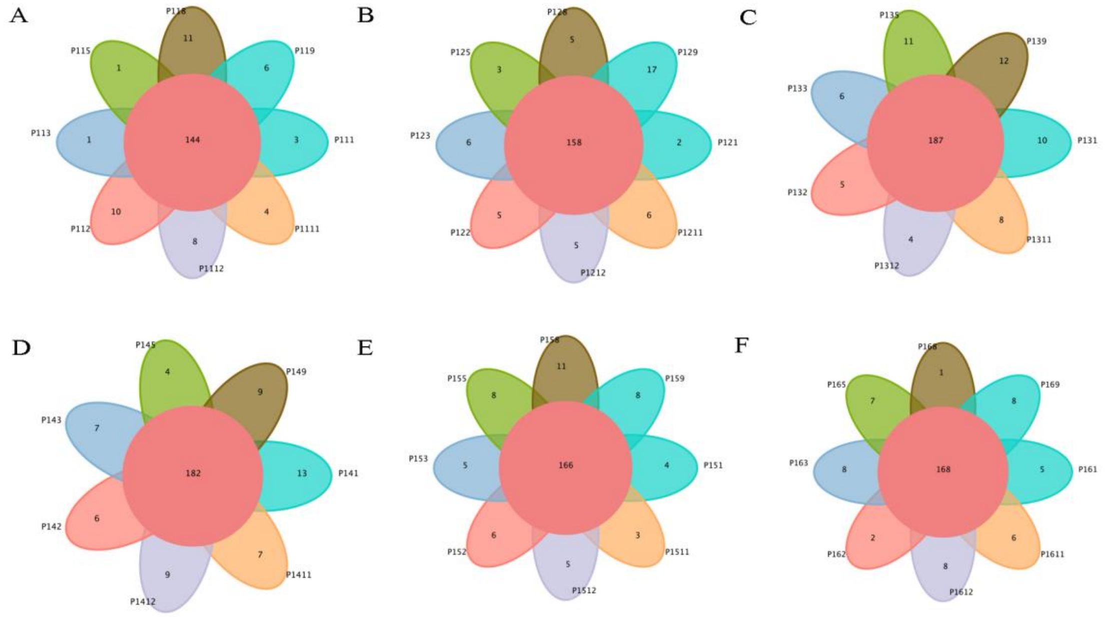

Figure S12. Prevalence of newborn rat visceral bacteria across organs (genera). Each organ possessed small numbers of exclusive bacteria with a low abundance compared to other organs. A to F represent six newborn rats. 1, brain; 2, heart; 3, liver; 4, spleen; 5, lung; 6, kidney; 7, pancreas; 8, skeletal muscle; 9, jejunum; 10, ileum; and 11, colon.

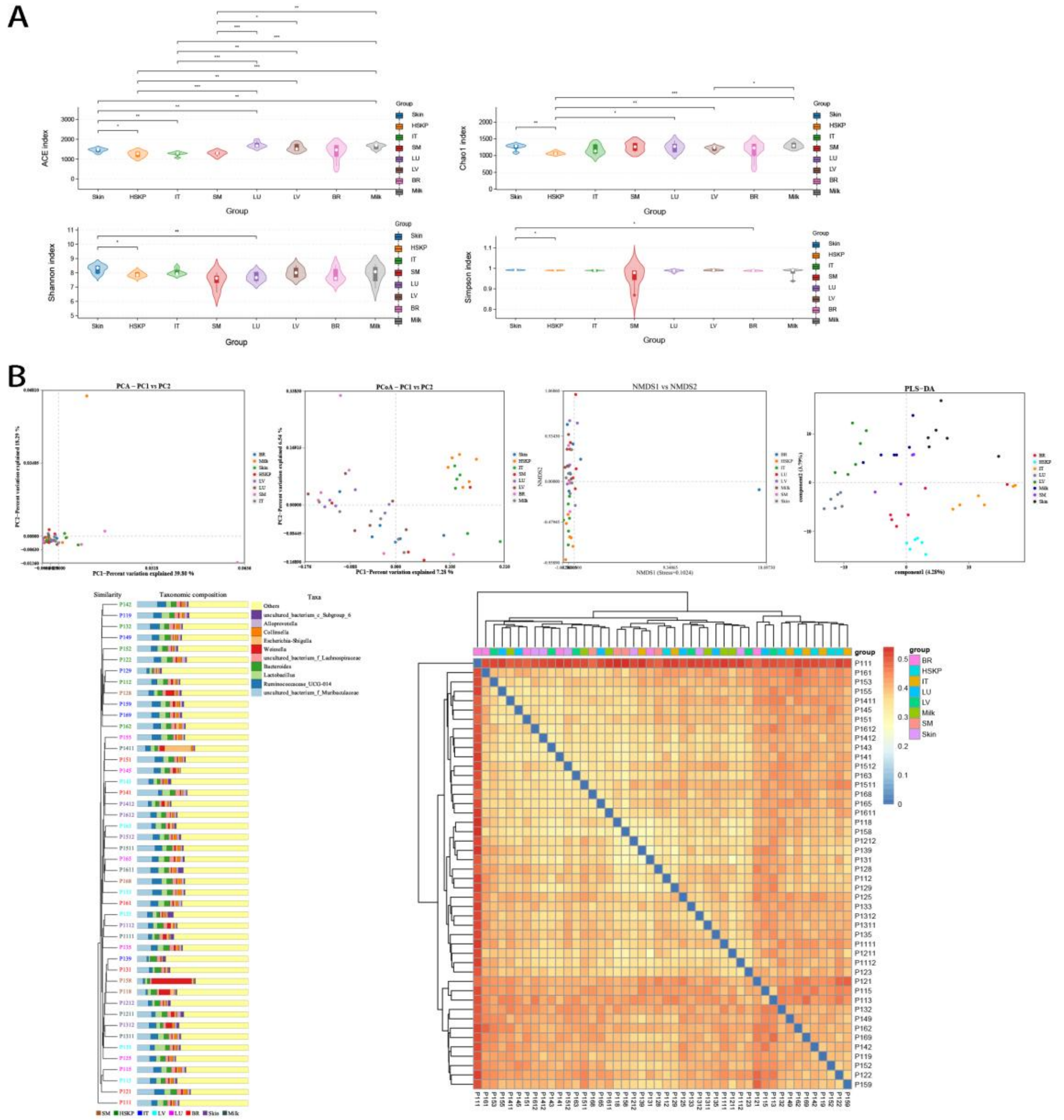

Figure S13. Diversity analysis of visceral bacteria in newborn rats (within 10 h after birth). (A) alpha diversity analysis. Simpson indices were calculated as $1-\sum p_i^2$. *$P < 0.05$; **$P < 0.01$; ***$P < 0.001$; and (B) beta diversity analysis. In the clustertree barplot and sample heatmap, brain (BR): P111-P161; heart, spleen, kidney, pancreas (HSKP): P112-P162; liver (LV): P113-P163; lung (LU): P115-P165; skeletal muscle (SM): P118-P168; intestinal tissue (IT): P119-P169; milk clot: P1111-P1611; skin: P1112-P1612.

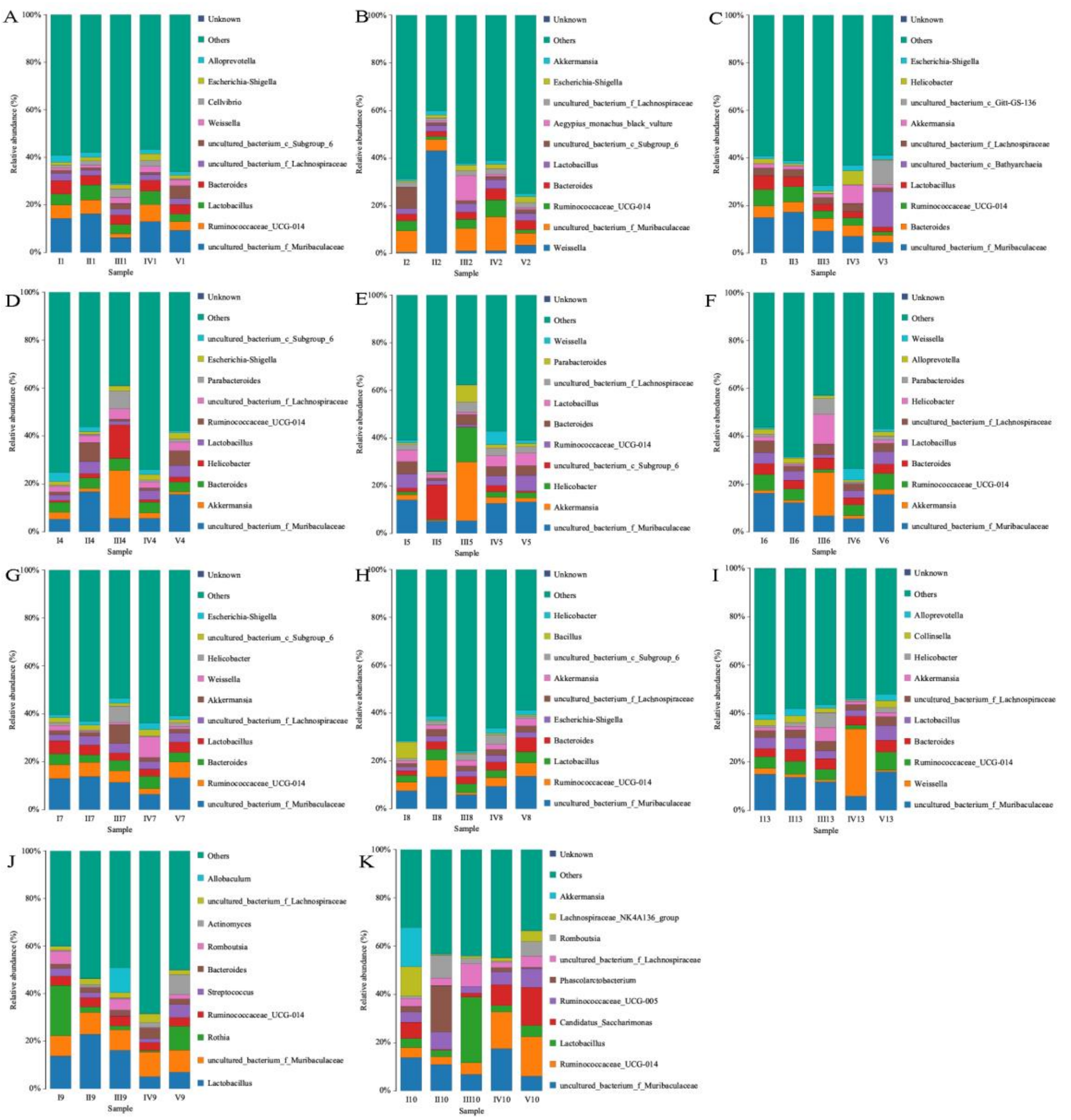

Figure S14. Variation of visceral bacteria across pregnant rats (genera, n = 5). A, brain; B, heart; C, liver; D, spleen; E, lung; F, kidney; G, pancreas; H, skeletal muscle; I, jejunum contents; J, ileum contents; and K, feces. Horizontal axes represent five pregnant rats.

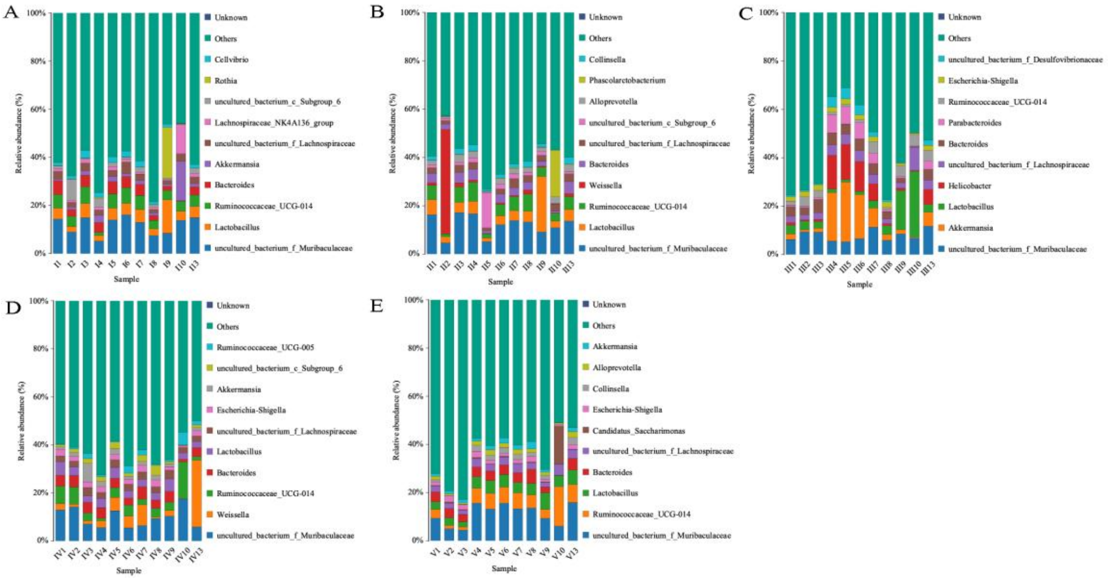

Figure S15. Variation of pregnant rat visceral bacteria across organs (genera). The visceral bacteria were constant across organs to some extent. A to E represent five pregnant rats. 1, brain; 2, heart; 3, liver; 4, spleen; 5, lung; 6, kidney; 7, pancreas; 8, skeletal muscle; 9, small intestine contents; 10, feces; and 13, mesenteric fat. Horizontal axes represent visceral organ and tissue and intestinal content.

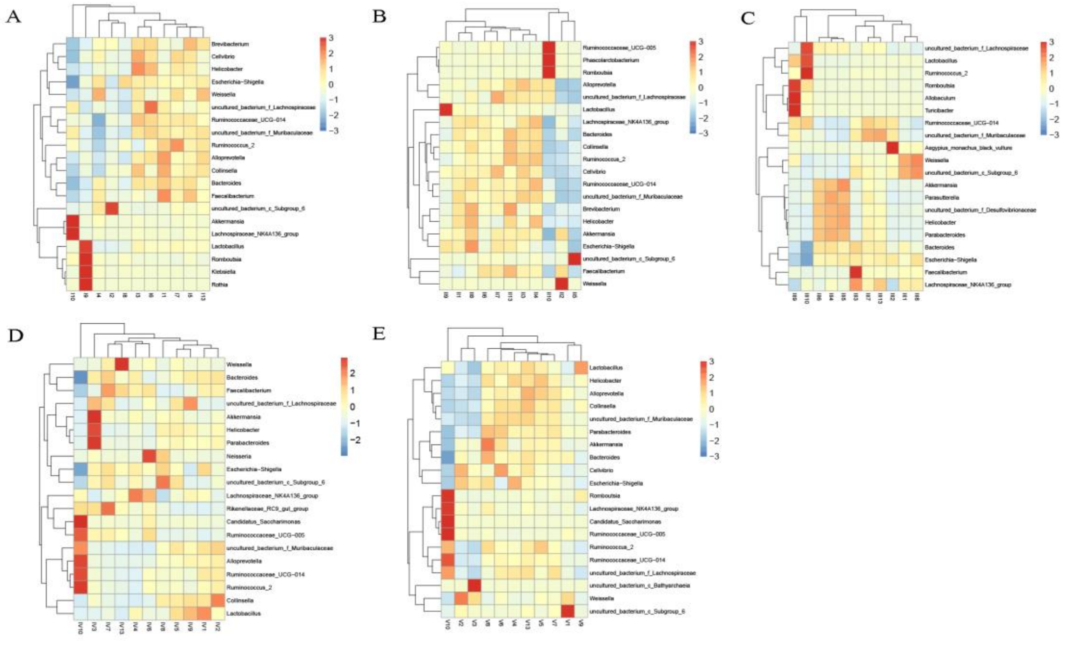

Figure S16. Relationship of visceral bacteria in an individual across organs (genera). A to F represent six rats. 1, brain; 2, heart; 3, liver; 4, spleen; 5, lung; 6, kidney; 7, pancreas; 8, skeletal muscle; 9, small intestine contents; 10, feces; and 13, mesenteric fat.

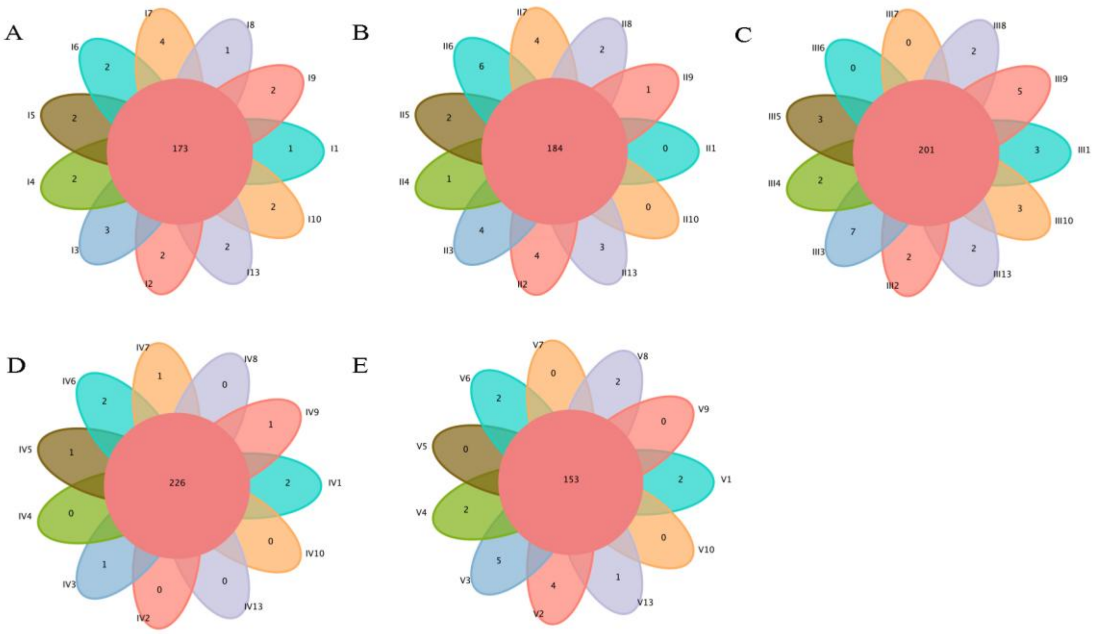

Figure S17. Prevalence of visceral bacteria of pregnant rats across organs (genera). Each organ possessed small numbers of exclusive bacteria with a low abundance compared to other organs. A to F represent six rats. 1, brain; 2, heart; 3, liver; 4, spleen; 5, lung; 6, kidney; 7, pancreas; 8, skeletal muscle; 9, small intestine contents; 10, feces; and 13, mesenteric fat.

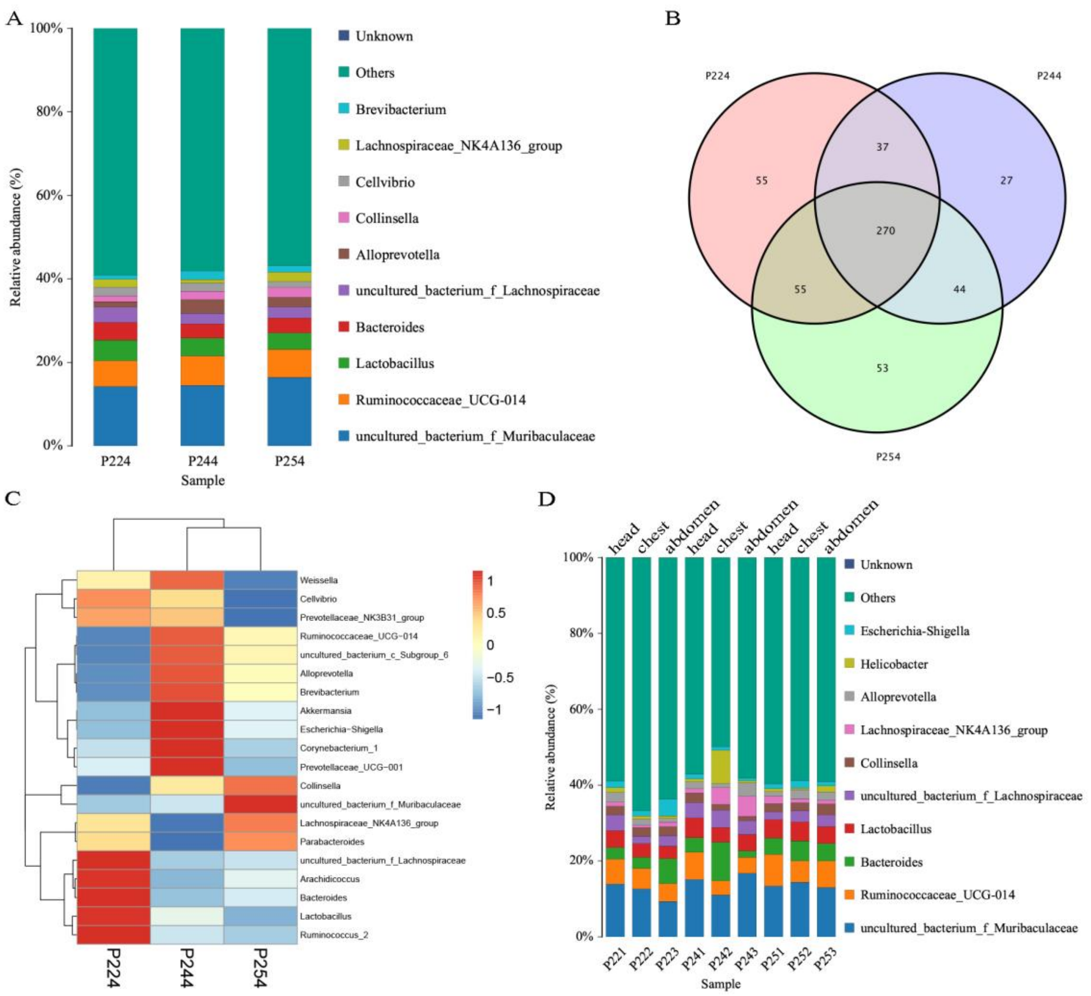

Figure S18. Bacteria composition of placentas and fetuses (genera). Placentas showed identical dominant bacteria (A), but each placenta contained exclusive bacteria (B and C). Fetuses from different pregnant rats showed relatively identical dominant bacteria compositions across organs and individuals (D), horizontal axes represent three fetus rats..

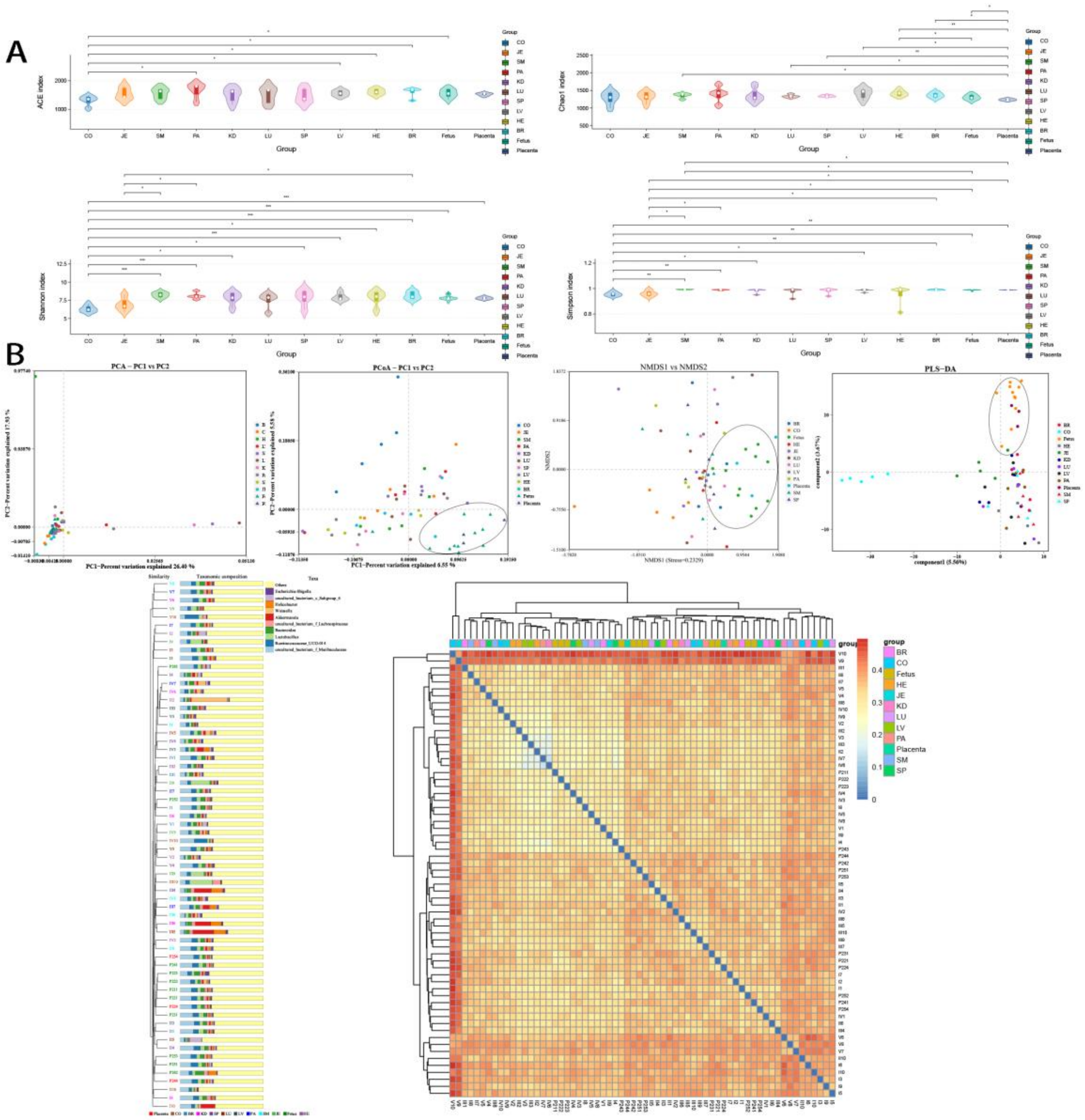

Figure S19. Diversity analysis of visceral bacteria of pregnant rats and their fetuses (n = 5). (A) Alpha diversity analysis. Simpson indices were calculated as $1-\sum p_i^2$. *P < 0.05; **P < 0.01; ***P < 0.001; (B) beta diversity analysis. Bacteria of fetuses and placentas are indicated in circles. In the clustertree barplot and sample heatmap, pregnant rats: I-V; 1, brain (BR); 2, heart (HE); 3, liver (LV); 4, spleen (SP); 5, lung (LU); 6, kidney (KD); 7, pancreas (PA); 8, skeletal muscle (SM); 9, jejunum (JE); and 10, colon (CO). Placenta: P224, p244, P254; and fetus: P211, P221-P223, P231, P241-243, P251-253.

Flow chart of the experiment

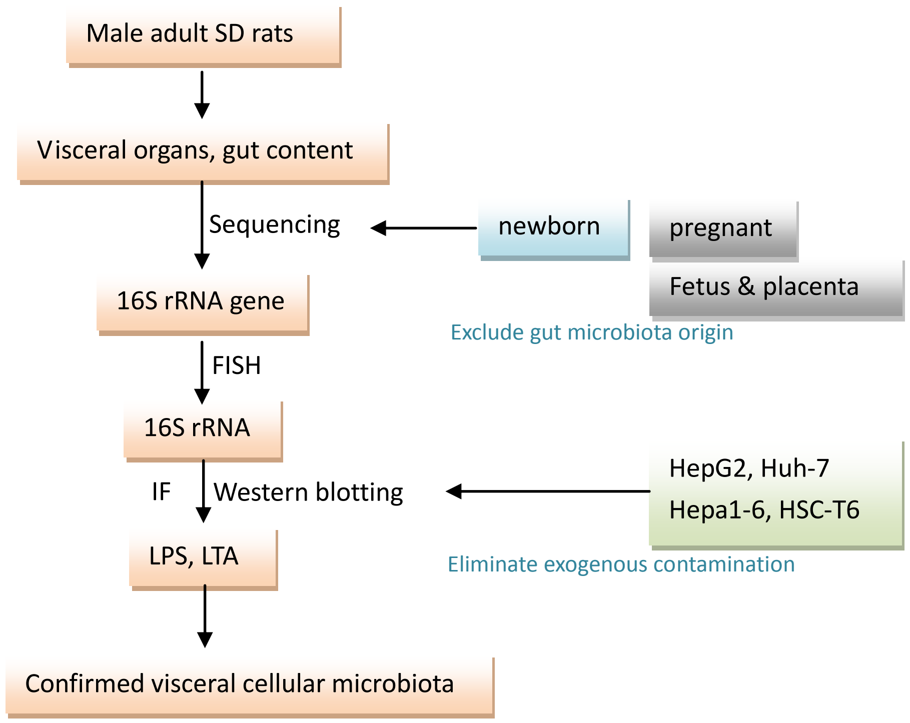

Figure legend

The 16S rRNA gene sequencing was performed in visceral organs of male adult SD rats (n = 6), pregnant rats (n = 6), newborn rats (n = 6, from the same litter), and fetuses and placentas (n = 6) to confirm the existence of visceral bacteria and to exclude the possible origin as gut microbiota. Fluorescence *in situ* hybridization (FISH) and immunofluorescence (IF) were performed *in situ* on visceral organs of male adult SD rats to detect the location of visceral bacteria. Western blotting was performed on nuclear and cytoplasmic extracts of visceral organs of SD rats and cell lines HepG2, Huh-7, Hepa1-6, and HSC-T6 to show the intrinsic inhabitant features and intracellular locations of the cellular bacteria.
