# Supplementary material for: The Cellular Microbiome of Visceral Organs: An Inherent Inhabitant of Parenchymal Cells
Source: Microorganisms. 2024 Jun 29;12(7):1333. doi: 10.3390/microorganisms12071333 (PMC11279389; doi:10.3390/microorganisms12071333)
Supplement: Supplementary file 1 [file microorganisms-12-01333-s001.zip › Supplementary Table S1.pdf]

Supplementary Table S1. Abundance and prevalence of annotated bacteria across visceral organs in male adult Sprague-Dawley rats.

Taxonomy	OTUs							
	SM	PA	KD	LU	SP	LV	HE	BR
Unassigned; Unassigned; Unassigned; Unassigned; Unassigned; Unassigned;	244	215	605	880	537	134	998	293
k__Bacteria; p__Acetothermia; c__Acetothermiia; o__uncultured_bacterium_c_Acetothermiia; f__uncultured_bacterium_c_Acetothermiia; g__uncultured_bacterium_c_Acetothermiia;	781	236	151	190	7	66	625	6
k__Bacteria; p__Acidobacteria; c__Acidobacteriia; o__Acidobacteriales; f__Acidobacteriaceae_Subgroup_1; g__Acidipila;	98	175	135	20	126	479	117	226
k__Bacteria; p__Acidobacteria; c__Acidobacteriia; o__Acidobacteriales; f__Acidobacteriaceae_Subgroup_1; g__Edaphobacter;	23	16	64	30	54	132	79	107
k__Bacteria; p__Acidobacteria; c__Acidobacteriia; o__Acidobacteriales; f__Acidobacteriaceae_Subgroup_1; g__Granulicella;	251	3	86	349	27	178	68	40
k__Bacteria; p__Acidobacteria; c__Acidobacteriia; o__Acidobacteriales; f__Acidobacteriaceae_Subgroup_1; g__Occallatibacter;	12	5	3	1	240	133	54	6
k__Bacteria; p__Acidobacteria; c__Acidobacteriia; o__Acidobacteriales; f__Acidobacteriaceae_Subgroup_1; g__uncultured_bacterium_f_Acidobacteriaceae_Subgroup_1;	1370	374	540	320	159	934	451	763
k__Bacteria; p__Acidobacteria; c__Acidobacteriia; o__Acidobacteriales; f__Koribacteraceae; g__Candidatus_Koribacter;	206	170	629	635	620	574	116	251
k__Bacteria; p__Acidobacteria; c__Acidobacteriia; o__Acidobacteriales; f__uncultured_bacterium_o_Acidobacteriales; g__uncultured_bacterium_o_Acidobacteriales;	1824	1465	2897	1777	1698	3233	2331	3260
k__Bacteria; p__Acidobacteria; c__Acidobacteriia; o__Solibacterales; f__Solibacteraceae_Subgroup_3; g__Bryobacter;	468	1051	1328	431	737	991	1472	505
k__Bacteria; p__Acidobacteria; c__Acidobacteriia; o__Solibacterales; f__Solibacteraceae_Subgroup_3; g__Candidatus_Solibacter;	897	531	1482	949	822	2174	805	1118
k__Bacteria; p__Acidobacteria; c__Acidobacteriia; o__Solibacterales; f__Solibacteraceae_Subgroup_3; g__PAUC26f;	219	0	0	17	0	0	0	6
k__Bacteria; p__Acidobacteria; c__Acidobacteriia; o__Solibacterales; f__Solibacteraceae_Subgroup_3; g__Paludibaculum;	153	472	346	69	118	5	0	12
k__Bacteria; p__Acidobacteria; c__Acidobacteriia; o__Solibacterales; f__Solibacteraceae_Subgroup_3; g__uncultured_bacterium_f_Solibacteraceae_Subgroup_3;	5	2	256	22	64	119	218	5
k__Bacteria; p__Acidobacteria; c__Acidobacteriia; o__Subgroup_13; f__uncultured_bacterium_o_Subgroup_13; g__uncultured_bacterium_o_Subgroup_13;	1	4	270	1	1	99	57	22
k__Bacteria; p__Acidobacteria; c__Acidobacteriia; o__Subgroup_2; f__uncultured_bacterium_o_Subgroup_2; g__uncultured_bacterium_o_Subgroup_2;	2070	1755	2239	1113	1965	4527	3145	2097
k__Bacteria; p__Acidobacteria; c__Aminicenantia; o__Aminicenantales; f__uncultured_bacterium_o_Aminicenantales; g__uncultured_bacterium_o_Aminicenantales;	10	17	372	33	177	59	172	100
k__Bacteria; p__Acidobacteria; c__Blastocatellia_Subgroup_4; o__11-24; f__uncultured_bacterium_o_11-24; g__uncultured_bacterium_o_11-24;	380	72	0	602	323	362	0	84
k__Bacteria; p__Acidobacteria; c__Blastocatellia_Subgroup_4; o__Blastocatellales; f__Blastocatellaceae; g__Stenotrophobacter;	6	1	27	47	39	58	0	3
k__Bacteria; p__Acidobacteria; c__Blastocatellia_Subgroup_4; o__Blastocatellales; f__Blastocatellaceae; g__uncultured_bacterium_f_Blastocatellaceae;	95	300	15	187	22	34	45	96
k__Bacteria; p__Acidobacteria; c__Blastocatellia_Subgroup_4; o__DS-100; f__uncultured_bacterium_o_DS-100; g__uncultured_bacterium_o_DS-100;	1	0	0	4	1	15	821	5
k__Bacteria; p__Acidobacteria; c__Blastocatellia_Subgroup_4; o__Pyrinomonadales; f__Pyrinomonadaceae; g__RB41;	59	48	380	72	139	407	138	143
k__Bacteria; p__Acidobacteria; c__Holophagae; o__Holophagales; f__Holophagaceae; g__Holophaga;	178	97	442	1	62	8	19	242

Taxonomy	OTUs							
	SM	PA	KD	LU	SP	LV	HE	BR
k__Bacteria; p__Acidobacteria; c__Holophagae; o__Subgroup_7; f__uncultured_bacterium_o_Subgroup_7; g__uncultured_bacterium_o_Subgroup_7;	671	145	341	211	451	424	481	427
k__Bacteria; p__Acidobacteria; c__Subgroup_17; o__uncultured_bacterium_c_Subgroup_17; f__uncultured_bacterium_c_Subgroup_17; g__uncultured_bacterium_c_Subgroup_17;	348	64	403	1383	449	394	152	69
k__Bacteria; p__Acidobacteria; c__Subgroup_18; o__uncultured_bacterium_c_Subgroup_18; f__uncultured_bacterium_c_Subgroup_18; g__uncultured_bacterium_c_Subgroup_18;	3	250	452	418	156	20	6	6
k__Bacteria; p__Acidobacteria; c__Subgroup_5; o__uncultured_bacterium_c_Subgroup_5; f__uncultured_bacterium_c_Subgroup_5; g__uncultured_bacterium_c_Subgroup_5;	51	33	108	67	168	102	66	37
k__Bacteria; p__Acidobacteria; c__Subgroup_6; o__Acidobacteria_bacterium_IGE-011; f__uncultured_bacterium_o_Acidobacteria_bacterium_IGE-011; g__uncultured_bacterium_o_Acidobacteria_bacterium_IGE-011;	125	102	477	61	66	253	136	194
k__Bacteria; p__Acidobacteria; c__Subgroup_6; o__uncultivated_soil_bacterium_clone_C028; f__uncultured_bacterium_o_uncultivated_soil_bacterium_clone_C028; g__uncultured_bacterium_o_uncultivated_soil_bacterium_clone_C028;	13	0	6	122	139	136	47	95
k__Bacteria; p__Acidobacteria; c__Subgroup_6; o__uncultivated_soil_bacterium_clone_C112; f__uncultured_bacterium_o_uncultivated_soil_bacterium_clone_C112; g__uncultured_bacterium_o_uncultivated_soil_bacterium_clone_C112;	204	333	246	622	10	550	200	260
*****k__Bacteria; p__Acidobacteria; c__Subgroup_6; o__uncultured_bacterium_c_Subgroup_6; f__uncultured_bacterium_c_Subgroup_6; g__uncultured_bacterium_c_Subgroup_6;	7368	3121	6430	6088	7335	10637	5010	7531
k__Bacteria; p__Acidobacteria; c__Subgroup_9; o__uncultured_bacterium_c_Subgroup_9; f__uncultured_bacterium_c_Subgroup_9; g__uncultured_bacterium_c_Subgroup_9;	1	0	97	139	2	5	27	1
k__Bacteria; p__Acidobacteria; c__Thermoanaerobaculia; o__Thermoanaerobaculales; f__Thermoanaerobaculaceae; g__Subgroup_10;	132	173	499	122	314	173	95	264
k__Bacteria; p__Actinobacteria; c__Acidimicrobiia; o__Actinomarinales; f__Actinomarinaceae; g__Candidatus_Actinomarina;	36	97	208	3	5	44	0	2
k__Bacteria; p__Actinobacteria; c__Acidimicrobiia; o__Actinomarinales; f__uncultured_bacterium_o_Actinomarinales; g__uncultured_bacterium_o_Actinomarinales;	69	1	1	236	0	21	0	47
k__Bacteria; p__Actinobacteria; c__Acidimicrobiia; o__IMCC26256; f__actinobacterium_BGR_88; g__uncultured_bacterium_f_actinobacterium_BGR_88;	671	220	113	37	253	23	29	72
k__Bacteria; p__Actinobacteria; c__Acidimicrobiia; o__IMCC26256; f__bacterium_enrichment_culture_clone_auto73_4W; g__uncultured_bacterium_f_bacterium_enrichment_culture_clone_auto73_4W;	206	390	210	80	3	2	1	386
k__Bacteria; p__Actinobacteria; c__Acidimicrobiia; o__IMCC26256; f__uncultured_bacterium_o_IMCC26256; g__uncultured_bacterium_o_IMCC26256;	405	87	727	343	322	705	323	459
k__Bacteria; p__Actinobacteria; c__Acidimicrobiia; o__Microtrichales; f__Iamiaceae; g__Iamia;	296	87	207	333	333	412	37	102
k__Bacteria; p__Actinobacteria; c__Acidimicrobiia; o__Microtrichales; f__Ilumatobacteraceae; g__CL500-29_marine_group;	36	145	1	80	202	306	3	333
k__Bacteria; p__Actinobacteria; c__Acidimicrobiia; o__Microtrichales; f__Ilumatobacteraceae; g__Ilumatobacter;	277	1	101	86	736	133	4	94
k__Bacteria; p__Actinobacteria; c__Acidimicrobiia; o__Microtrichales; f__Ilumatobacteraceae; g__uncultured_bacterium_f_Ilumatobacteraceae;	61	1	81	506	206	202	2	308

Taxonomy	OTUs							
	SM	PA	KD	LU	SP	LV	HE	BR
k__Bacteria; p__Actinobacteria; c__Acidimicrobiia; o__Microtrichales; f__uncultured_bacterium_o_Microtrichales; g__uncultured_bacterium_o_Microtrichales;	204	230	126	132	600	45	26	154
k__Bacteria; p__Actinobacteria; c__Acidimicrobiia; o__uncultured_bacterium_c_Acidimicrobiia; f__uncultured_bacterium_c_Acidimicrobiia; g__uncultured_bacterium_c_Acidimicrobiia;	29	417	686	269	145	263	440	514
k__Bacteria; p__Actinobacteria; c__Actinobacteria; o__Actinomycetales; f__Actinomycetaceae; g__Actinomyces;	180	400	322	240	199	208	421	109
k__Bacteria; p__Actinobacteria; c__Actinobacteria; o__Bifidobacteriales; f__Bifidobacteriaceae; g__Bifidobacterium;	740	1401	486	523	645	1110	973	651
k__Bacteria; p__Actinobacteria; c__Actinobacteria; o__Bifidobacteriales; f__Bifidobacteriaceae; g__Gardnerella;	7	68	201	11	98	134	73	22
k__Bacteria; p__Actinobacteria; c__Actinobacteria; o__Catenulisporales; f__Actinospicaceae; g__Actinospica;	13	149	186	3	8	74	53	26
k__Bacteria; p__Actinobacteria; c__Actinobacteria; o__Catenulisporales; f__Catenulisporaceae; g__Catenulispora;	41	4	279	6	110	3	146	4
k__Bacteria; p__Actinobacteria; c__Actinobacteria; o__Corynebacteriales; f__Corynebacteriaceae; g__Corynebacterium;	2	29	251	169	95	271	163	71
k__Bacteria; p__Actinobacteria; c__Actinobacteria; o__Corynebacteriales; f__Corynebacteriaceae; g__Corynebacterium_1;	4747	4537	3931	3843	4307	3022	5461	3734
k__Bacteria; p__Actinobacteria; c__Actinobacteria; o__Corynebacteriales; f__Corynebacteriaceae; g__Lawsonella;	792	550	528	695	1096	717	863	425
k__Bacteria; p__Actinobacteria; c__Actinobacteria; o__Corynebacteriales; f__Corynebacteriaceae; g__uncultured_bacterium_f_Corynebacteriaceae;	28	114	147	56	83	2	112	4
*****k__Bacteria; p__Actinobacteria; c__Actinobacteria; o__Corynebacteriales; f__Dietziaceae; g__Dietzia;	4726	15003	13475	16072	23693	7743	11901	7239
k__Bacteria; p__Actinobacteria; c__Actinobacteria; o__Corynebacteriales; f__Mycobacteriaceae; g__Mycobacterium;	170	518	347	375	65	634	175	290
k__Bacteria; p__Actinobacteria; c__Actinobacteria; o__Corynebacteriales; f__Nocardiaceae; g__Gordonia;	2	184	3	295	128	160	138	15
k__Bacteria; p__Actinobacteria; c__Actinobacteria; o__Corynebacteriales; f__Nocardiaceae; g__Nocardia;	0	0	47	0	104	44	0	20
k__Bacteria; p__Actinobacteria; c__Actinobacteria; o__Corynebacteriales; f__Nocardiaceae; g__Rhodococcus;	3568	4607	3937	3563	5955	2045	5713	2313
k__Bacteria; p__Actinobacteria; c__Actinobacteria; o__Corynebacteriales; f__Tsukamurellaceae; g__Tsukamurella;	0	60	130	6	18	0	14	30
k__Bacteria; p__Actinobacteria; c__Actinobacteria; o__Frankiales; f__Acidothermaceae; g__Acidothermus;	122	215	503	257	201	1282	974	714
k__Bacteria; p__Actinobacteria; c__Actinobacteria; o__Frankiales; f__Frankiaceae; g__Jatrophihabitans;	57	109	43	122	242	41	41	51
k__Bacteria; p__Actinobacteria; c__Actinobacteria; o__Frankiales; f__Geodermatophilaceae; g__Blastococcus;	49	52	9	148	854	628	55	536
k__Bacteria; p__Actinobacteria; c__Actinobacteria; o__Frankiales; f__Geodermatophilaceae; g__Geodermatophilus;	87	4	0	100	9	93	8	147
k__Bacteria; p__Actinobacteria; c__Actinobacteria; o__Frankiales; f__Geodermatophilaceae; g__Modestobacter;	321	58	129	12	5	27	3	155
k__Bacteria; p__Actinobacteria; c__Actinobacteria; o__Frankiales; f__Nakamurellaceae; g__Nakamurella;	2	0	0	249	2	8	52	41

Taxonomy	OTUs							
	SM	PA	KD	LU	SP	LV	HE	BR
k__Bacteria; p__Actinobacteria; c__Actinobacteria; o__Frankiales; f__uncultured_bacterium_o_Frankiales; g__uncultured_bacterium_o_Frankiales;	52	246	36	41	520	469	191	260
k__Bacteria; p__Actinobacteria; c__Actinobacteria; o__Glycomycetales; f__Glycomycetaceae; g__Glycomyces;	58	2	99	78	28	0	0	19
k__Bacteria; p__Actinobacteria; c__Actinobacteria; o__Kineosporiales; f__Kineosporiaceae; g__Pseudokineococcus;	241	121	280	15	23	6	4	7
k__Bacteria; p__Actinobacteria; c__Actinobacteria; o__Kineosporiales; f__Kineosporiaceae; g__Quadrisphaera;	0	1216	2	3	101	101	2	26
k__Bacteria; p__Actinobacteria; c__Actinobacteria; o__Micrococcales; f__Bogoriellaceae; g__Bogoriella;	2	16	38	50	5	126	305	0
k__Bacteria; p__Actinobacteria; c__Actinobacteria; o__Micrococcales; f__Bogoriellaceae; g__uncultured_bacterium_f_Bogoriellaceae;	896	118	597	408	330	165	1539	387
*****k__Bacteria; p__Actinobacteria; c__Actinobacteria; o__Micrococcales; f__Brevibacteriaceae; g__Brevibacterium;	6462	5421	5936	5324	8873	3741	8756	7022
k__Bacteria; p__Actinobacteria; c__Actinobacteria; o__Micrococcales; f__Cellulomonadaceae; g__Cellulomonas;	13	5	93	232	361	196	144	184
k__Bacteria; p__Actinobacteria; c__Actinobacteria; o__Micrococcales; f__Demequinaceae; g__uncultured_bacterium_f_Demequinaceae;	147	245	1	81	328	197	2	2
k__Bacteria; p__Actinobacteria; c__Actinobacteria; o__Micrococcales; f__Dermabacteraceae; g__Brachybacterium;	286	581	998	232	985	278	402	802
k__Bacteria; p__Actinobacteria; c__Actinobacteria; o__Micrococcales; f__Dermabacteraceae; g__Dermabacter;	0	0	0	69	0	5	262	0
k__Bacteria; p__Actinobacteria; c__Actinobacteria; o__Micrococcales; f__Dermacoccaceae; g__Kytococcus;	131	63	76	29	47	194	1	1
k__Bacteria; p__Actinobacteria; c__Actinobacteria; o__Micrococcales; f__Intrasporangiaceae; g__uncultured_bacterium_f_Intrasporangiaceae;	613	162	140	596	462	584	445	1034
k__Bacteria; p__Actinobacteria; c__Actinobacteria; o__Micrococcales; f__Microbacteriaceae; g__Agromyces;	34	13	170	172	11	59	1	17
k__Bacteria; p__Actinobacteria; c__Actinobacteria; o__Micrococcales; f__Microbacteriaceae; g__Microbacterium;	4	102	17	14	10	8	106	9
k__Bacteria; p__Actinobacteria; c__Actinobacteria; o__Micrococcales; f__Microbacteriaceae; g__uncultured_bacterium_f_Microbacteriaceae;	358	286	308	527	908	386	70	320
k__Bacteria; p__Actinobacteria; c__Actinobacteria; o__Micrococcales; f__Micrococcaceae; g__Arthrobacter;	323	9	71	425	125	98	347	529
k__Bacteria; p__Actinobacteria; c__Actinobacteria; o__Micrococcales; f__Micrococcaceae; g__Enteractinococcus;	11	83	43	125	232	41	57	1
k__Bacteria; p__Actinobacteria; c__Actinobacteria; o__Micrococcales; f__Micrococcaceae; g__Haematomicrobium;	1	27	193	218	62	2	113	0
k__Bacteria; p__Actinobacteria; c__Actinobacteria; o__Micrococcales; f__Micrococcaceae; g__Nesterenkonia;	567	1231	432	936	1712	464	914	299
k__Bacteria; p__Actinobacteria; c__Actinobacteria; o__Micrococcales; f__Micrococcaceae; g__Paenarthrobacter;	122	0	0	23	89	0	79	0
k__Bacteria; p__Actinobacteria; c__Actinobacteria; o__Micrococcales; f__Micrococcaceae; g__Rothia;	454	555	173	202	415	338	184	353
k__Bacteria; p__Actinobacteria; c__Actinobacteria; o__Micrococcales; f__Micrococcaceae; g__Sinomonas;	52	12	360	53	18	8	5	13
k__Bacteria; p__Actinobacteria; c__Actinobacteria; o__Micrococcales; f__Micrococcaceae; g__uncultured_bacterium_f_Micrococcaceae;	2185	2306	1950	1296	1124	2759	2205	3988

Taxonomy	OTUs							
	SM	PA	KD	LU	SP	LV	HE	BR
k__Bacteria; p__Actinobacteria; c__Actinobacteria; o__Micrococcales; f__Promicromonosporaceae; g__Cellulosimicrobium;	7	0	2	577	65	24	3	25
k__Bacteria; p__Actinobacteria; c__Actinobacteria; o__Micrococcales; f__Promicromonosporaceae; g__Promicromonospora;	2	2	246	99	1	280	14	42
k__Bacteria; p__Actinobacteria; c__Actinobacteria; o__Micromonosporales; f__Micromonosporaceae; g__Micromonospora;	110	26	54	99	422	153	204	339
k__Bacteria; p__Actinobacteria; c__Actinobacteria; o__Micromonosporales; f__Micromonosporaceae; g__uncultured_bacterium_f_Micromonosporaceae;	0	0	0	1	0	337	0	0
k__Bacteria; p__Actinobacteria; c__Actinobacteria; o__PeM15; f__uncultured_bacterium_o_PeM15; g__uncultured_bacterium_o_PeM15;	10	165	0	17	3	9	29	355
k__Bacteria; p__Actinobacteria; c__Actinobacteria; o__Propionibacteriales; f__Nocardioidaceae; g__Kribbella;	0	1	120	2	0	3	1	228
k__Bacteria; p__Actinobacteria; c__Actinobacteria; o__Propionibacteriales; f__Nocardioidaceae; g__Marmoricola;	167	285	163	69	144	375	192	305
k__Bacteria; p__Actinobacteria; c__Actinobacteria; o__Propionibacteriales; f__Nocardioidaceae; g__Nocardioides;	21	51	70	281	340	861	273	170
k__Bacteria; p__Actinobacteria; c__Actinobacteria; o__Propionibacteriales; f__Propionibacteriaceae; g__Cutibacterium;	105	63	77	73	82	47	68	68
k__Bacteria; p__Actinobacteria; c__Actinobacteria; o__Pseudonocardiales; f__Pseudonocardiaceae; g__Actinophytocola;	2	0	116	20	9	84	4	9
k__Bacteria; p__Actinobacteria; c__Actinobacteria; o__Pseudonocardiales; f__Pseudonocardiaceae; g__Amycolatopsis;	208	34	173	20	18	135	8	103
k__Bacteria; p__Actinobacteria; c__Actinobacteria; o__Pseudonocardiales; f__Pseudonocardiaceae; g__Pseudonocardia;	565	427	525	586	1191	724	794	216
k__Bacteria; p__Actinobacteria; c__Actinobacteria; o__Pseudonocardiales; f__Pseudonocardiaceae; g__Saccharomonospora;	98	164	223	37	230	81	0	7
k__Bacteria; p__Actinobacteria; c__Actinobacteria; o__Pseudonocardiales; f__Pseudonocardiaceae; g__Saccharothrix;	0	1	153	138	1	283	2	10
k__Bacteria; p__Actinobacteria; c__Actinobacteria; o__Pseudonocardiales; f__Pseudonocardiaceae; g__uncultured_bacterium_f_Pseudonocardiaceae;	131	39	35	4	177	261	0	99
k__Bacteria; p__Actinobacteria; c__Actinobacteria; o__Streptomycetales; f__Streptomycetaceae; g__Streptomyces;	27	398	204	656	186	293	202	661
k__Bacteria; p__Actinobacteria; c__Actinobacteria; o__Streptomycetales; f__Streptomycetaceae; g__uncultured_bacterium_f_Streptomycetaceae;	930	399	646	838	394	795	233	328
k__Bacteria; p__Actinobacteria; c__Actinobacteria; o__Streptosporangiales; f__Nocardiopsaceae; g__Nocardiopsis;	930	571	694	28	968	1713	518	401
k__Bacteria; p__Actinobacteria; c__Actinobacteria; o__Streptosporangiales; f__Nocardiopsaceae; g__Streptomonospora;	25	37	107	2	37	143	1	0
k__Bacteria; p__Actinobacteria; c__Actinobacteria; o__Streptosporangiales; f__Streptosporangiaceae; g__Microbispora;	0	0	1	162	1	1	86	2
k__Bacteria; p__Actinobacteria; c__Coriobacteriia; o__Coriobacteriales; f__Atopobiaceae; g__Coriobacteriaceae_UCG-002;	136	259	367	44	13	6	385	27
k__Bacteria; p__Actinobacteria; c__Coriobacteriia; o__Coriobacteriales; f__Atopobiaceae; g__Olsenella;	139	1	246	2	98	58	42	16
k__Bacteria; p__Actinobacteria; c__Coriobacteriia; o__Coriobacteriales; f__Atopobiaceae; g__uncultured_bacterium_f_Atopobiaceae;	297	369	47	605	145	18	221	8
k__Bacteria; p__Actinobacteria; c__Coriobacteriia; o__Coriobacteriales; f__Coriobacteriaceae; g__Collinsella;	69	71	7	16	45	187	67	88

Taxonomy	OTUs							
	SM	PA	KD	LU	SP	LV	HE	BR
k__Bacteria; p__Actinobacteria; c__Coriobacteriia; o__Coriobacteriales; f__Eggerthellaceae; g__Adlercreutzia;	49	5	4	0	1	1	3	15
k__Bacteria; p__Actinobacteria; c__Coriobacteriia; o__Coriobacteriales; f__Eggerthellaceae; g__DNF00809;	0	103	0	0	135	0	0	0
k__Bacteria; p__Actinobacteria; c__Coriobacteriia; o__Coriobacteriales; f__Eggerthellaceae; g__Eggerthella;	365	0	126	76	3	161	3	0
k__Bacteria; p__Actinobacteria; c__Coriobacteriia; o__Coriobacteriales; f__Eggerthellaceae; g__Enterorhabdus;	43	349	140	8	64	348	95	366
k__Bacteria; p__Actinobacteria; c__Coriobacteriia; o__Coriobacteriales; f__Eggerthellaceae; g__uncultured_bacterium_f_Eggerthellaceae;	22	91	0	5	0	146	0	0
k__Bacteria; p__Actinobacteria; c__MB-A2-108; o__uncultured_bacterium_c_MB-A2-108; f__uncultured_bacterium_c_MB-A2-108; g__uncultured_bacterium_c_MB-A2-108;	165	527	640	303	613	714	205	866
k__Bacteria; p__Actinobacteria; c__Nitriliruptoria; o__Nitriliruptorales; f__Nitriliruptoraceae; g__uncultured_bacterium_f_Nitriliruptoraceae;	1563	5750	4211	5569	7761	2358	5677	3150
k__Bacteria; p__Actinobacteria; c__Thermoleophilia; o__Gaiellales; f__Gaiellaceae; g__Gaiella;	143	296	572	244	262	292	170	598
k__Bacteria; p__Actinobacteria; c__Thermoleophilia; o__Gaiellales; f__uncultured_bacterium_o_Gaiellales; g__uncultured_bacterium_o_Gaiellales;	1072	615	995	330	884	861	1157	1487
k__Bacteria; p__Actinobacteria; c__Thermoleophilia; o__Solirubrobacterales; f__67-14; g__uncultured_bacterium_f_67-14;	4	0	81	115	109	42	49	65
k__Bacteria; p__Actinobacteria; c__Thermoleophilia; o__Solirubrobacterales; f__Solirubrobacteraceae; g__Solirubrobacter;	142	84	69	66	104	299	149	221
k__Bacteria; p__Actinobacteria; c__Thermoleophilia; o__Solirubrobacterales; f__Solirubrobacteraceae; g__uncultured_bacterium_f_Solirubrobacteraceae;	3	80	2	4	147	70	64	29
k__Bacteria; p__Armatimonadetes; c__Chthonomonadetes; o__Chthonomonadales; f__uncultured_bacterium_o_Chthonomonadales; g__uncultured_bacterium_o_Chthonomonadales;	1	0	0	1	254	0	0	1
k__Bacteria; p__Bacteroidetes; c__Bacteroidia; o__Bacteroidales; f__Bacteroidaceae; g__Bacteroides;	3606	2544	2418	2361	4584	3685	4296	3076
k__Bacteria; p__Bacteroidetes; c__Bacteroidia; o__Bacteroidales; f__Bacteroidales_BS11_gut_group; g__uncultured_bacterium_f_Bacteroidales_BS11_gut_group;	44	121	31	0	95	0	54	0
k__Bacteria; p__Bacteroidetes; c__Bacteroidia; o__Bacteroidales; f__Bacteroidales_RF16_group; g__uncultured_bacterium_f_Bacteroidales_RF16_group;	0	0	0	0	1	1	0	258
k__Bacteria; p__Bacteroidetes; c__Bacteroidia; o__Bacteroidales; f__Bacteroidetes_vadinHA17; g__uncultured_bacterium_f_Bacteroidetes_vadinHA17;	81	108	415	64	199	203	283	145
k__Bacteria; p__Bacteroidetes; c__Bacteroidia; o__Bacteroidales; f__F082; g__uncultured_bacterium_f_F082;	0	341	393	2	0	0	0	2
k__Bacteria; p__Bacteroidetes; c__Bacteroidia; o__Bacteroidales; f__Muribaculaceae; g__Gram-negative_bacterium_cTPY-13;	360	78	47	55	46	26	51	166
k__Bacteria; p__Bacteroidetes; c__Bacteroidia; o__Bacteroidales; f__Muribaculaceae; g__uncultured_bacterium_f_Muribaculaceae;	2594	5198	1292	2128	1393	1490	1781	2875
k__Bacteria; p__Bacteroidetes; c__Bacteroidia; o__Bacteroidales; f__Porphyromonadaceae; g__Porphyromonas;	3	4	3	111	0	128	3	32
k__Bacteria; p__Bacteroidetes; c__Bacteroidia; o__Bacteroidales; f__Prevotellaceae; g__Alloprevotella;	26	155	157	20	17	39	31	26
k__Bacteria; p__Bacteroidetes; c__Bacteroidia; o__Bacteroidales; f__Prevotellaceae; g__Prevotella_1;	298	201	279	5	219	816	226	3
k__Bacteria; p__Bacteroidetes; c__Bacteroidia; o__Bacteroidales; f__Prevotellaceae; g__Prevotella_7;	34	0	0	1	4	9	216	37

Taxonomy	OTUs							
	SM	PA	KD	LU	SP	LV	HE	BR
k__Bacteria; p__Bacteroidetes; c__Bacteroidia; o__Bacteroidales; f__Prevotellaceae; g__Prevotella_9;	339	735	189	470	411	353	446	387
k__Bacteria; p__Bacteroidetes; c__Bacteroidia; o__Bacteroidales; f__Prevotellaceae; g__Prevotellaceae_NK3B31_group;	676	150	244	375	479	54	79	273
k__Bacteria; p__Bacteroidetes; c__Bacteroidia; o__Bacteroidales; f__Prevotellaceae; g__Prevotellaceae_UCG-001;	105	140	38	202	90	31	29	89
k__Bacteria; p__Bacteroidetes; c__Bacteroidia; o__Bacteroidales; f__Prevotellaceae; g__Prevotellaceae_UCG-003;	152	2	73	41	25	49	3	22
k__Bacteria; p__Bacteroidetes; c__Bacteroidia; o__Bacteroidales; f__Prevotellaceae; g__uncultured_bacterium_f_Prevotellaceae;	1056	243	195	97	505	88	275	1400
k__Bacteria; p__Bacteroidetes; c__Bacteroidia; o__Bacteroidales; f__Rikenellaceae; g__Alistipes;	101	198	50	133	362	248	106	184
k__Bacteria; p__Bacteroidetes; c__Bacteroidia; o__Bacteroidales; f__Rikenellaceae; g__Rikenellaceae_RC9_gut_group;	130	125	23	18	141	12	54	251
k__Bacteria; p__Bacteroidetes; c__Bacteroidia; o__Bacteroidales; f__Tannerellaceae; g__Parabacteroides;	183	336	16	3	28	24	68	39
k__Bacteria; p__Bacteroidetes; c__Bacteroidia; o__Bacteroidales; f__uncultured_bacterium_o_Bacteroidales; g__uncultured_bacterium_o_Bacteroidales;	0	0	13	1	2	0	710	1
k__Bacteria; p__Bacteroidetes; c__Bacteroidia; o__Chitinophagales; f__Chitinophagaceae; g__Sediminibacterium;	364	322	266	522	221	80	605	302
k__Bacteria; p__Bacteroidetes; c__Bacteroidia; o__Chitinophagales; f__Chitinophagaceae; g__Taibaiella;	181	2	0	0	0	54	2	16
k__Bacteria; p__Bacteroidetes; c__Bacteroidia; o__Chitinophagales; f__Chitinophagaceae; g__uncultured_bacterium_f_Chitinophagaceae;	186	22	2	24	0	3	369	148
k__Bacteria; p__Bacteroidetes; c__Bacteroidia; o__Chitinophagales; f__Saprospiraceae; g__uncultured_bacterium_f_Saprospiraceae;	222	2	4	0	0	0	1	112
k__Bacteria; p__Bacteroidetes; c__Bacteroidia; o__Cytophagales; f__Cyclobacteriaceae; g__uncultured_bacterium_f_Cyclobacteriaceae;	15	137	3	28	136	143	33	17
k__Bacteria; p__Bacteroidetes; c__Bacteroidia; o__Cytophagales; f__Hymenobacteraceae; g__Adhaeribacter;	1	0	4	1	0	2028	11	6
k__Bacteria; p__Bacteroidetes; c__Bacteroidia; o__Cytophagales; f__Hymenobacteraceae; g__Hymenobacter;	1	1212	2	1	2	155	4	2
k__Bacteria; p__Bacteroidetes; c__Bacteroidia; o__Cytophagales; f__Microscillaceae; g__Chryseolinea;	98	2	55	217	124	2	117	224
k__Bacteria; p__Bacteroidetes; c__Bacteroidia; o__Cytophagales; f__Microscillaceae; g__Ohtaekwangia;	19	0	0	182	1	35	2	0
k__Bacteria; p__Bacteroidetes; c__Bacteroidia; o__Cytophagales; f__Microscillaceae; g__uncultured_bacterium_f_Microscillaceae;	614	150	591	230	238	698	118	184
k__Bacteria; p__Bacteroidetes; c__Bacteroidia; o__Cytophagales; f__Spirosomaceae; g__Dyadobacter;	13	194	11	23	22	1	16	101
k__Bacteria; p__Bacteroidetes; c__Bacteroidia; o__Cytophagales; f__Spirosomaceae; g__Flectobacillus;	80	1	232	0	0	0	0	1
k__Bacteria; p__Bacteroidetes; c__Bacteroidia; o__Cytophagales; f__Spirosomaceae; g__Leadbetterella;	51	104	70	163	0	174	93	114
k__Bacteria; p__Bacteroidetes; c__Bacteroidia; o__Cytophagales; f__Spirosomaceae; g__Persicitalea;	1	2	57	48	1	94	61	35
k__Bacteria; p__Bacteroidetes; c__Bacteroidia; o__Cytophagales; f__Spirosomaceae; g__Spirosoma;	0	0	0	0	0	0	0	463

Taxonomy	OTUs							
	SM	PA	KD	LU	SP	LV	HE	BR
k__Bacteria; p__Bacteroidetes; c__Bacteroidia; o__Flavobacteriales; f__Blattabacteriaceae; g__Candidatus_Sulcia;	6	9	17	86	28	29	35	22
k__Bacteria; p__Bacteroidetes; c__Bacteroidia; o__Flavobacteriales; f__Weeksellaceae; g__Chryseobacterium;	137	259	251	139	218	3	264	273
k__Bacteria; p__Bacteroidetes; c__Bacteroidia; o__Flavobacteriales; f__Weeksellaceae; g__Cloacibacterium;	69	51	107	155	150	286	10	19
k__Bacteria; p__Bacteroidetes; c__Bacteroidia; o__Sphingobacteriales; f__Sphingobacteriaceae; g__Mucilaginibacter;	112	6	1	81	75	0	0	4
k__Bacteria; p__Bacteroidetes; c__Bacteroidia; o__uncultured_bacterium_c_Bacteroidia; f__uncultured_bacterium_c_Bacteroidia; g__uncultured_bacterium_c_Bacteroidia;	0	0	0	0	2	0	1	280
k__Bacteria; p__Bacteroidetes; c__Rhodothermia; o__Balneolales; f__Balneolaceae; g__uncultured_bacterium_f_Balneolaceae;	21	232	83	136	457	76	228	13
k__Bacteria; p__Caldiserica; c__Caldisericia; o__Caldisericales; f__Caldisericaceae; g__Caldisericum;	1	4	196	119	322	176	40	3
k__Bacteria; p__Chloroflexi; c__AD3; o__uncultured_bacterium_c_AD3; f__uncultured_bacterium_c_AD3; g__uncultured_bacterium_c_AD3;	89	481	283	68	50	155	826	274
k__Bacteria; p__Chloroflexi; c__Anaerolineae; o__Anaerolineales; f__Anaerolineaceae; g__ADurb.Bin120;	0	290	193	4	0	78	1	331
k__Bacteria; p__Chloroflexi; c__Anaerolineae; o__Anaerolineales; f__Anaerolineaceae; g__Anaerolinea;	20	0	0	1	378	49	259	207
k__Bacteria; p__Chloroflexi; c__Anaerolineae; o__Anaerolineales; f__Anaerolineaceae; g__Bellilinea;	1	1	0	303	1	0	0	0
k__Bacteria; p__Chloroflexi; c__Anaerolineae; o__Anaerolineales; f__Anaerolineaceae; g__RBG-16-58-14;	108	293	47	170	172	54	296	386
k__Bacteria; p__Chloroflexi; c__Anaerolineae; o__Anaerolineales; f__Anaerolineaceae; g__uncultured_bacterium_f_Anaerolineaceae;	478	389	1043	1074	1324	211	910	195
k__Bacteria; p__Chloroflexi; c__Anaerolineae; o__RBG-13-54-9; f__uncultured_bacterium_o_RBG-13-54-9; g__uncultured_bacterium_o_RBG-13-54-9;	1	1	0	207	0	6	69	0
k__Bacteria; p__Chloroflexi; c__Anaerolineae; o__SBR1031; f__A4b; g__uncultured_bacterium_f_A4b;	0	0	119	26	62	2	196	173
k__Bacteria; p__Chloroflexi; c__Anaerolineae; o__SBR1031; f__uncultured_bacterium_o_SBR1031; g__uncultured_bacterium_o_SBR1031;	69	27	206	2	144	230	61	29
k__Bacteria; p__Chloroflexi; c__Gitt-GS-136; o__uncultured_bacterium_c_Gitt-GS-136; f__uncultured_bacterium_c_Gitt-GS-136; g__uncultured_bacterium_c_Gitt-GS-136;	285	89	49	178	1	35	132	63
k__Bacteria; p__Chloroflexi; c__JG30-KF-CM66; o__uncultured_bacterium_c_JG30-KF-CM66; f__uncultured_bacterium_c_JG30-KF-CM66; g__uncultured_bacterium_c_JG30-KF-CM66;	2	2	237	0	2	0	246	104
k__Bacteria; p__Chloroflexi; c__KD4-96; o__uncultured_bacterium_c_KD4-96; f__uncultured_bacterium_c_KD4-96; g__uncultured_bacterium_c_KD4-96;	309	94	203	89	436	380	63	484
k__Bacteria; p__Chloroflexi; c__Ktedonobacteria; o__Ktedonobacterales; f__JG30-KF-AS9; g__uncultured_bacterium_f_JG30-KF-AS9;	262	62	6	219	327	231	300	247
k__Bacteria; p__Chloroflexi; c__uncultured_bacterium_p_Chloroflexi; o__uncultured_bacterium_p_Chloroflexi; f__uncultured_bacterium_p_Chloroflexi; g__uncultured_bacterium_p_Chloroflexi;	0	2	0	0	442	0	3	1
k__Bacteria; p__Cyanobacteria; c__Melainabacteria; o__Gastranaerophilales; f__uncultured_bacterium_o_Gastranaerophilales; g__uncultured_bacterium_o_Gastranaerophilales;	444	93	29	11	134	89	264	109
k__Bacteria; p__Cyanobacteria; c__Melainabacteria; o__Obscuribacterales; f__uncultured_bacterium_o_Obscuribacterales; g__uncultured_bacterium_o_Obscuribacterales;	517	370	633	1208	862	622	582	816

Taxonomy	OTUs							
	SM	PA	KD	LU	SP	LV	HE	BR
k__Bacteria; p__Cyanobacteria; c__Oxyphotobacteria; o__Chloroplast; f__Desmochloris_halophila; g__Desmochloris_halophila;	10	3823	7	3	10	382	7	10
k__Bacteria; p__Cyanobacteria; c__Oxyphotobacteria; o__Chloroplast; f__Nephrolepis_biserrata_var._furcans; g__Nephrolepis_biserrata_var._furcans;	0	0	196	1	6	0	0	24
k__Bacteria; p__Cyanobacteria; c__Oxyphotobacteria; o__Chloroplast; f__Spirogyra_maxima; g__Spirogyra_maxima;	2	1	55	3	108	115	183	35
k__Bacteria; p__Cyanobacteria; c__Oxyphotobacteria; o__Chloroplast; f__Trachydiscus_minutus; g__Trachydiscus_minutus;	210	6	0	1	171	0	130	0
k__Bacteria; p__Cyanobacteria; c__Oxyphotobacteria; o__Chloroplast; f__uncultured_bacterium_o_Chloroplast; g__uncultured_bacterium_o_Chloroplast;	2883	3365	2840	3870	4059	6046	6558	3570
k__Bacteria; p__Cyanobacteria; c__Oxyphotobacteria; o__Nostocales; f__Chroococcidiopsaceae; g__Aliterella_CENA595;	124	618	159	46	46	134	463	35
k__Bacteria; p__Cyanobacteria; c__Oxyphotobacteria; o__Nostocales; f__Chroococcidiopsaceae; g__Chroococcidiopsis_PCC_7203;	765	742	1409	1525	947	978	1083	1570
k__Bacteria; p__Cyanobacteria; c__Oxyphotobacteria; o__Nostocales; f__Chroococcidiopsaceae; g__Chroococcidiopsis_SAG_2023;	66	364	2	0	1	6	0	0
k__Bacteria; p__Cyanobacteria; c__Oxyphotobacteria; o__Nostocales; f__Chroococcidiopsaceae; g__uncultured_bacterium_f_Chroococcidiopsaceae;	0	2	305	0	0	0	2	1
k__Bacteria; p__Cyanobacteria; c__Oxyphotobacteria; o__Nostocales; f__Microcystaceae; g__Pleurocapsa_PCC-7327;	0	1	290	3	0	158	0	181
k__Bacteria; p__Cyanobacteria; c__Oxyphotobacteria; o__Nostocales; f__uncultured_bacterium_o_Nostocales; g__uncultured_bacterium_o_Nostocales;	8	2961	6	7	11	339	0	1
k__Bacteria; p__Cyanobacteria; c__Oxyphotobacteria; o__Synechococcales; f__Cyanobiaceae; g__Synechococcus_CC9902;	0	54	2	18	4	164	10	80
k__Bacteria; p__Deinococcus-Thermus; c__Deinococci; o__Deinococcales; f__Trueperaceae; g__Truepera;	388	1	2	1	1	11	1	0
k__Bacteria; p__Dependentiae; c__Babeliae; o__Babeliales; f__uncultured_bacterium_o_Babeliales; g__uncultured_bacterium_o_Babeliales;	0	0	0	0	1	0	246	0
k__Bacteria; p__Elusimicrobia; c__Elusimicrobia; o__Elusimicrobiales; f__Elusimicrobiaceae; g__Elusimicrobium;	1	0	280	2	71	71	1	3
k__Bacteria; p__Elusimicrobia; c__Elusimicrobia; o__MVP-88; f__uncultured_bacterium_o_MVP-88; g__uncultured_bacterium_o_MVP-88;	11	0	328	1	2	1	23	228
k__Bacteria; p__Elusimicrobia; c__Endomicrobia; o__Endomicrobiales; f__Endomicrobiaceae; g__Candidatus_Endomicrobium;	1	1	0	289	0	0	1	0
k__Bacteria; p__Elusimicrobia; c__Lineage_IIa; o__uncultured_bacterium_c_Lineage_IIa; f__uncultured_bacterium_c_Lineage_IIa; g__uncultured_bacterium_c_Lineage_IIa;	12	0	2	1	0	276	1	3
k__Bacteria; p__Epsilonbacteraeota; c__Campylobacteria; o__Campylobacterales; f__Arcobacteraceae; g__Arcobacter;	356	4	260	177	914	631	160	2
k__Bacteria; p__Epsilonbacteraeota; c__Campylobacteria; o__Campylobacterales; f__Campylobacteraceae; g__Campylobacter;	78	206	72	14	1026	232	276	22
k__Bacteria; p__Epsilonbacteraeota; c__Campylobacteria; o__Campylobacterales; f__Helicobacteraceae; g__Helicobacter;	606	649	674	195	668	1659	842	6754
k__Bacteria; p__Epsilonbacteraeota; c__Campylobacteria; o__Campylobacterales; f__Sulfurospirillaceae; g__Sulfurospirillum;	3	0	0	4	1	0	295	1
k__Bacteria; p__Epsilonbacteraeota; c__Campylobacteria; o__Campylobacterales; f__Thiovulaceae; g__Sulfuricurvum;	0	2	66	80	38	17	2	109
k__Bacteria; p__Epsilonbacteraeota; c__Campylobacteria; o__Campylobacterales; f__Thiovulaceae; g__Sulfurimonas;	236	292	408	354	112	950	466	4

Taxonomy	OTUs							
	SM	PA	KD	LU	SP	LV	HE	BR
k__Bacteria; p__Firmicutes; c__Bacilli; o__Bacillales; f__Alicyclobacillaceae; g__Tumebacillus;	18	45	50	583	273	13	3	305
k__Bacteria; p__Firmicutes; c__Bacilli; o__Bacillales; f__Bacillaceae; g__Aeribacillus;	1	11	401	1	1	245	169	164
k__Bacteria; p__Firmicutes; c__Bacilli; o__Bacillales; f__Bacillaceae; g__Bacillus;	3252	3313	3838	8862	4229	4175	4628	5033
k__Bacteria; p__Firmicutes; c__Bacilli; o__Bacillales; f__Bacillaceae; g__Geobacillus;	21	57	100	3	8	0	0	90
k__Bacteria; p__Firmicutes; c__Bacilli; o__Bacillales; f__Bacillaceae; g__Oceanobacillus;	0	92	0	2	255	348	0	0
k__Bacteria; p__Firmicutes; c__Bacilli; o__Bacillales; f__Bacillaceae; g__Ureibacillus;	5	0	35	2	55	192	143	32
k__Bacteria; p__Firmicutes; c__Bacilli; o__Bacillales; f__Bacillaceae; g__Virgibacillus;	34	90	127	36	174	204	205	532
k__Bacteria; p__Firmicutes; c__Bacilli; o__Bacillales; f__Bacillaceae; g__uncultured_bacterium_f_Bacillaceae;	25	232	338	287	379	87	472	98
k__Bacteria; p__Firmicutes; c__Bacilli; o__Bacillales; f__Family_XI; g__Gemella;	196	231	278	214	181	370	132	218
k__Bacteria; p__Firmicutes; c__Bacilli; o__Bacillales; f__Family_XII; g__Exiguobacterium;	60	10	233	251	27	280	39	126
k__Bacteria; p__Firmicutes; c__Bacilli; o__Bacillales; f__Paenibacillaceae; g__Aneurinibacillus;	34	144	166	40	3	354	228	132
k__Bacteria; p__Firmicutes; c__Bacilli; o__Bacillales; f__Paenibacillaceae; g__Brevibacillus;	2	50	1	34	30	35	505	328
k__Bacteria; p__Firmicutes; c__Bacilli; o__Bacillales; f__Paenibacillaceae; g__Paenibacillus;	110	429	57	588	1137	435	1092	402
k__Bacteria; p__Firmicutes; c__Bacilli; o__Bacillales; f__Planococcaceae; g__Domibacillus;	360	3	0	31	3	49	1	2
k__Bacteria; p__Firmicutes; c__Bacilli; o__Bacillales; f__Planococcaceae; g__Kurthia;	159	159	282	325	125	293	310	22
k__Bacteria; p__Firmicutes; c__Bacilli; o__Bacillales; f__Planococcaceae; g__Lysinibacillus;	75	36	65	104	63	119	61	120
k__Bacteria; p__Firmicutes; c__Bacilli; o__Bacillales; f__Planococcaceae; g__Paenisporosarcina;	60	76	0	15	3	133	0	39
k__Bacteria; p__Firmicutes; c__Bacilli; o__Bacillales; f__Sporolactobacillaceae; g__Alkalicoccus;	5	165	124	28	127	153	29	1
k__Bacteria; p__Firmicutes; c__Bacilli; o__Bacillales; f__Staphylococcaceae; g__Jeotgalicoccus;	88	247	241	70	252	13	612	75
k__Bacteria; p__Firmicutes; c__Bacilli; o__Bacillales; f__Staphylococcaceae; g__Nosocomiicoccus;	175	404	284	201	119	69	124	30
k__Bacteria; p__Firmicutes; c__Bacilli; o__Bacillales; f__Staphylococcaceae; g__S31;	13	11	2	2	0	47	40	91
k__Bacteria; p__Firmicutes; c__Bacilli; o__Bacillales; f__Staphylococcaceae; g__Staphylococcus;	4646	4870	2679	4218	6545	2598	4980	4867
k__Bacteria; p__Firmicutes; c__Bacilli; o__Bacillales; f__Thermoactinomycetaceae; g__uncultured_bacterium_f_Thermoactinomycetaceae;	21	0	19	0	0	125	150	0
k__Bacteria; p__Firmicutes; c__Bacilli; o__Lactobacillales; f__Aerococcaceae; g__Aerococcus;	49	6	2	249	3	167	60	234

Taxonomy	OTUs							
	SM	PA	KD	LU	SP	LV	HE	BR
k__Bacteria; p__Firmicutes; c__Bacilli; o__Lactobacillales; f__Aerococcaceae; g__Facklamia;	4	126	3	10	9	4	3	8
k__Bacteria; p__Firmicutes; c__Bacilli; o__Lactobacillales; f__Aerococcaceae; g__Globicatella;	0	364	2	98	14	1	2	4
k__Bacteria; p__Firmicutes; c__Bacilli; o__Lactobacillales; f__Carnobacteriaceae; g__Atopostipes;	27	132	0	66	1	5	2	3
k__Bacteria; p__Firmicutes; c__Bacilli; o__Lactobacillales; f__Carnobacteriaceae; g__Dolosigranulum;	6	77	86	92	0	0	0	2
k__Bacteria; p__Firmicutes; c__Bacilli; o__Lactobacillales; f__Carnobacteriaceae; g__Trichococcus;	23	120	126	15	3	28	51	20
k__Bacteria; p__Firmicutes; c__Bacilli; o__Lactobacillales; f__Enterococcaceae; g__Enterococcus;	628	157	621	218	308	878	43	695
*****k__Bacteria; p__Firmicutes; c__Bacilli; o__Lactobacillales; f__Lactobacillaceae; g__Lactobacillus;	18127	35734	8531	11064	8578	10267	11686	11948
k__Bacteria; p__Firmicutes; c__Bacilli; o__Lactobacillales; f__Lactobacillaceae; g__Pediococcus;	386	98	12	1	30	64	216	27
k__Bacteria; p__Firmicutes; c__Bacilli; o__Lactobacillales; f__Leuconostocaceae; g__Leuconostoc;	4	6	7	4	2	180	80	7
k__Bacteria; p__Firmicutes; c__Bacilli; o__Lactobacillales; f__Leuconostocaceae; g__Weissella;	191	282	506	10	145	153	311	98
k__Bacteria; p__Firmicutes; c__Bacilli; o__Lactobacillales; f__Streptococcaceae; g__Lactococcus;	125	228	235	177	321	424	278	199
k__Bacteria; p__Firmicutes; c__Bacilli; o__Lactobacillales; f__Streptococcaceae; g__Streptococcus;	3072	5899	3305	5052	3707	3391	3106	2826
k__Bacteria; p__Firmicutes; c__Bacilli; o__Lactobacillales; f__uncultured_bacterium_o_Lactobacillales; g__uncultured_bacterium_o_Lactobacillales;	276	8	45	103	109	237	18	53
k__Bacteria; p__Firmicutes; c__Clostridia; o__Clostridiales; f__Christensenellaceae; g__Christensenellaceae_R-7_group;	412	190	487	85	199	415	276	338
k__Bacteria; p__Firmicutes; c__Clostridia; o__Clostridiales; f__Christensenellaceae; g__uncultured_bacterium_f_Christensenellaceae;	103	41	159	2	20	198	226	5
k__Bacteria; p__Firmicutes; c__Clostridia; o__Clostridiales; f__Clostridiaceae_1; g__Candidatus_Arthromitus;	97	231	31	502	345	160	125	369
k__Bacteria; p__Firmicutes; c__Clostridia; o__Clostridiales; f__Clostridiaceae_1; g__Clostridium_sensu_stricto_11;	0	2	2	35	1	0	1	200
k__Bacteria; p__Firmicutes; c__Clostridia; o__Clostridiales; f__Clostridiaceae_1; g__Clostridium_sensu_stricto_13;	1	4	62	3	1	317	18	24
k__Bacteria; p__Firmicutes; c__Clostridia; o__Clostridiales; f__Clostridiaceae_1; g__Clostridium_sensu_stricto_1;	827	630	1332	142	589	1397	832	236
k__Bacteria; p__Firmicutes; c__Clostridia; o__Clostridiales; f__Clostridiales_vadinBB60_group; g__uncultured_bacterium_f_Clostridiales_vadinBB60_group;	230	45	281	7	155	309	189	5
k__Bacteria; p__Firmicutes; c__Clostridia; o__Clostridiales; f__Defluviitaleaceae; g__Defluviitaleaceae_UCG-011;	0	1	221	0	0	0	0	0
k__Bacteria; p__Firmicutes; c__Clostridia; o__Clostridiales; f__Family_XI; g__Anaerococcus;	151	238	700	1007	483	172	470	283
k__Bacteria; p__Firmicutes; c__Clostridia; o__Clostridiales; f__Family_XI; g__Finegoldia;	129	72	23	171	86	0	242	97

Taxonomy	OTUs							
	SM	PA	KD	LU	SP	LV	HE	BR
k__Bacteria; p__Firmicutes; c__Clostridia; o__Clostridiales; f__Family_XI; g__Peptoniphilus;	610	383	251	458	552	323	629	331
k__Bacteria; p__Firmicutes; c__Clostridia; o__Clostridiales; f__Family_XIII; g__Family_XIII_UCG-001;	165	28	4	0	0	0	1	0
k__Bacteria; p__Firmicutes; c__Clostridia; o__Clostridiales; f__Family_XIII; g__[Eubacterium]_brachy_group;	0	0	33	0	1	13	1	271
k__Bacteria; p__Firmicutes; c__Clostridia; o__Clostridiales; f__Family_XIII; g__[Eubacterium]_nodatum_group;	178	24	44	20	1	4	29	0
k__Bacteria; p__Firmicutes; c__Clostridia; o__Clostridiales; f__Family_XIII; g__uncultured_bacterium_f_Family_XIII;	351	4	0	220	4	2	3	3
k__Bacteria; p__Firmicutes; c__Clostridia; o__Clostridiales; f__Lachnospiraceae; g__A2;	0	2	0	219	1	158	1	0
k__Bacteria; p__Firmicutes; c__Clostridia; o__Clostridiales; f__Lachnospiraceae; g__Anaerocolumna;	1	1	15	2	251	92	1	0
k__Bacteria; p__Firmicutes; c__Clostridia; o__Clostridiales; f__Lachnospiraceae; g__Anaerostipes;	46	69	6	70	321	101	22	78
k__Bacteria; p__Firmicutes; c__Clostridia; o__Clostridiales; f__Lachnospiraceae; g__Blautia;	3373	2131	1710	2078	1389	2878	2808	1860
k__Bacteria; p__Firmicutes; c__Clostridia; o__Clostridiales; f__Lachnospiraceae; g__Coprococcus_2;	0	81	0	3	133	1	21	0
k__Bacteria; p__Firmicutes; c__Clostridia; o__Clostridiales; f__Lachnospiraceae; g__Dorea;	54	298	114	24	122	142	59	130
k__Bacteria; p__Firmicutes; c__Clostridia; o__Clostridiales; f__Lachnospiraceae; g__Epulopiscium;	91	0	1	2	1	0	77	36
k__Bacteria; p__Firmicutes; c__Clostridia; o__Clostridiales; f__Lachnospiraceae; g__Fusicatenibacter;	924	280	355	100	98	288	364	1583
k__Bacteria; p__Firmicutes; c__Clostridia; o__Clostridiales; f__Lachnospiraceae; g__GCA-900066575;	69	35	0	27	3	1	2	6
k__Bacteria; p__Firmicutes; c__Clostridia; o__Clostridiales; f__Lachnospiraceae; g__Johnsonella;	79	0	1	4	274	67	0	4
k__Bacteria; p__Firmicutes; c__Clostridia; o__Clostridiales; f__Lachnospiraceae; g__Lachnoclostridium;	492	979	896	872	1186	875	1533	1309
k__Bacteria; p__Firmicutes; c__Clostridia; o__Clostridiales; f__Lachnospiraceae; g__Lachnospira;	397	9	64	237	7	9	43	578
k__Bacteria; p__Firmicutes; c__Clostridia; o__Clostridiales; f__Lachnospiraceae; g__Lachnospiraceae_FCS020_group;	192	0	1	2	1	0	1	0
k__Bacteria; p__Firmicutes; c__Clostridia; o__Clostridiales; f__Lachnospiraceae; g__Lachnospiraceae_ND3007_group;	352	519	350	180	229	239	104	208
k__Bacteria; p__Firmicutes; c__Clostridia; o__Clostridiales; f__Lachnospiraceae; g__Lachnospiraceae_NK4A136_group;	1729	1086	1381	3384	811	1281	507	930
k__Bacteria; p__Firmicutes; c__Clostridia; o__Clostridiales; f__Lachnospiraceae; g__Lachnospiraceae_UCG-001;	13	16	29	7	70	0	84	13
k__Bacteria; p__Firmicutes; c__Clostridia; o__Clostridiales; f__Lachnospiraceae; g__Lachnospiraceae_UCG-004;	51	152	8	157	314	240	131	19
k__Bacteria; p__Firmicutes; c__Clostridia; o__Clostridiales; f__Lachnospiraceae; g__Lachnospiraceae_UCG-010;	59	122	3	121	83	60	26	26
k__Bacteria; p__Firmicutes; c__Clostridia; o__Clostridiales; f__Lachnospiraceae; g__Pseudobutyrivibrio;	171	168	93	27	121	893	247	18

Taxonomy	OTUs							
	SM	PA	KD	LU	SP	LV	HE	BR
k__Bacteria; p__Firmicutes; c__Clostridia; o__Clostridiales; f__Lachnospiraceae; g__Roseburia;	2814	1849	1766	436	1468	965	1110	1839
k__Bacteria; p__Firmicutes; c__Clostridia; o__Clostridiales; f__Lachnospiraceae; g__Sellimonas;	26	61	0	0	231	29	50	0
k__Bacteria; p__Firmicutes; c__Clostridia; o__Clostridiales; f__Lachnospiraceae; g__Shuttleworthia;	9	4	4	4	50	141	2	3
k__Bacteria; p__Firmicutes; c__Clostridia; o__Clostridiales; f__Lachnospiraceae; g__Tyzzerella;	66	1	35	26	6	48	65	80
k__Bacteria; p__Firmicutes; c__Clostridia; o__Clostridiales; f__Lachnospiraceae; g__Tyzzerella_3;	382	238	0	365	4	25	3	420
k__Bacteria; p__Firmicutes; c__Clostridia; o__Clostridiales; f__Lachnospiraceae; g__Tyzzerella_4;	3	10	1	2	5	152	16	5
k__Bacteria; p__Firmicutes; c__Clostridia; o__Clostridiales; f__Lachnospiraceae; g__[Eubacterium]_eligens_group;	196	11	183	10	309	63	219	306
k__Bacteria; p__Firmicutes; c__Clostridia; o__Clostridiales; f__Lachnospiraceae; g__[Eubacterium]_hallii_group;	91	147	3	143	7	186	41	54
k__Bacteria; p__Firmicutes; c__Clostridia; o__Clostridiales; f__Lachnospiraceae; g__[Eubacterium]_oxidoreducens_group;	1	0	0	0	0	0	0	366
k__Bacteria; p__Firmicutes; c__Clostridia; o__Clostridiales; f__Lachnospiraceae; g__[Eubacterium]_ruminantium_group;	92	76	27	1	0	117	12	1
k__Bacteria; p__Firmicutes; c__Clostridia; o__Clostridiales; f__Lachnospiraceae; g__[Eubacterium]_ventriosum_group;	109	31	170	3	1	38	0	11
k__Bacteria; p__Firmicutes; c__Clostridia; o__Clostridiales; f__Lachnospiraceae; g__[Eubacterium]_xylanophilum_group;	523	563	358	498	34	32	223	444
k__Bacteria; p__Firmicutes; c__Clostridia; o__Clostridiales; f__Lachnospiraceae; g__[Ruminococcus]_gnavus_group;	304	427	402	257	516	706	862	161
k__Bacteria; p__Firmicutes; c__Clostridia; o__Clostridiales; f__Lachnospiraceae; g__[Ruminococcus]_torques_group;	595	336	432	338	941	1837	871	298
k__Bacteria; p__Firmicutes; c__Clostridia; o__Clostridiales; f__Lachnospiraceae; g__uncultured_bacterium_f_Lachnospiraceae;	8172	5112	7521	3803	4513	3014	5291	7611
k__Bacteria; p__Firmicutes; c__Clostridia; o__Clostridiales; f__Peptococcaceae; g__uncultured_bacterium_f_Peptococcaceae;	111	56	48	10	83	19	80	64
k__Bacteria; p__Firmicutes; c__Clostridia; o__Clostridiales; f__Peptostreptococcaceae; g__Intestinibacter;	60	2	3	2	61	74	29	49
k__Bacteria; p__Firmicutes; c__Clostridia; o__Clostridiales; f__Peptostreptococcaceae; g__Romboutsia;	3362	3056	1023	943	1149	922	2255	1494
k__Bacteria; p__Firmicutes; c__Clostridia; o__Clostridiales; f__Peptostreptococcaceae; g__Terrisporobacter;	49	343	52	5	47	367	207	65
k__Bacteria; p__Firmicutes; c__Clostridia; o__Clostridiales; f__Peptostreptococcaceae; g__uncultured_bacterium_f_Peptostreptococcaceae;	0	0	0	0	290	1	2	0
k__Bacteria; p__Firmicutes; c__Clostridia; o__Clostridiales; f__Ruminococcaceae; g__Anaerotruncus;	1	1	145	2	87	0	0	0
k__Bacteria; p__Firmicutes; c__Clostridia; o__Clostridiales; f__Ruminococcaceae; g__Butyricicoccus;	68	51	143	5	2	31	132	43
k__Bacteria; p__Firmicutes; c__Clostridia; o__Clostridiales; f__Ruminococcaceae; g__DTU089;	0	1	169	3	41	170	102	0
k__Bacteria; p__Firmicutes; c__Clostridia; o__Clostridiales; f__Ruminococcaceae; g__Faecalibacterium;	901	433	2068	316	1968	1860	779	382

Taxonomy	OTUs							
	SM	PA	KD	LU	SP	LV	HE	BR
k__Bacteria; p__Firmicutes; c__Clostridia; o__Clostridiales; f__Ruminococcaceae; g__Flavonifractor;	1	5	67	145	3	89	2	3
k__Bacteria; p__Firmicutes; c__Clostridia; o__Clostridiales; f__Ruminococcaceae; g__Fournierella;	0	56	105	3	22	0	87	3
k__Bacteria; p__Firmicutes; c__Clostridia; o__Clostridiales; f__Ruminococcaceae; g__Hydrogenoanaerobacterium;	0	2	157	2	1	114	24	1
k__Bacteria; p__Firmicutes; c__Clostridia; o__Clostridiales; f__Ruminococcaceae; g__Intestinimonas;	1	39	4	8	5	29	145	27
k__Bacteria; p__Firmicutes; c__Clostridia; o__Clostridiales; f__Ruminococcaceae; g__Negativibacillus;	92	245	132	81	2	210	247	27
k__Bacteria; p__Firmicutes; c__Clostridia; o__Clostridiales; f__Ruminococcaceae; g__Oscillibacter;	1124	295	194	64	32	30	27	731
k__Bacteria; p__Firmicutes; c__Clostridia; o__Clostridiales; f__Ruminococcaceae; g__Pygmaiobacter;	105	1	38	9	1	0	1	102
k__Bacteria; p__Firmicutes; c__Clostridia; o__Clostridiales; f__Ruminococcaceae; g__Ruminiclostridium;	110	63	14	68	4	0	155	7
k__Bacteria; p__Firmicutes; c__Clostridia; o__Clostridiales; f__Ruminococcaceae; g__Ruminiclostridium_5;	14	143	136	23	3	223	41	12
k__Bacteria; p__Firmicutes; c__Clostridia; o__Clostridiales; f__Ruminococcaceae; g__Ruminiclostridium_6;	973	329	210	144	61	202	401	547
k__Bacteria; p__Firmicutes; c__Clostridia; o__Clostridiales; f__Ruminococcaceae; g__Ruminiclostridium_9;	840	962	629	155	323	547	421	343
k__Bacteria; p__Firmicutes; c__Clostridia; o__Clostridiales; f__Ruminococcaceae; g__Ruminococcaceae_NK4A214_group;	292	168	259	75	378	354	418	111
k__Bacteria; p__Firmicutes; c__Clostridia; o__Clostridiales; f__Ruminococcaceae; g__Ruminococcaceae_UCG-002;	134	245	520	116	53	108	114	79
k__Bacteria; p__Firmicutes; c__Clostridia; o__Clostridiales; f__Ruminococcaceae; g__Ruminococcaceae_UCG-004;	243	0	4	2	0	1	1	31
k__Bacteria; p__Firmicutes; c__Clostridia; o__Clostridiales; f__Ruminococcaceae; g__Ruminococcaceae_UCG-005;	792	190	550	671	1100	723	655	1131
k__Bacteria; p__Firmicutes; c__Clostridia; o__Clostridiales; f__Ruminococcaceae; g__Ruminococcaceae_UCG-008;	484	93	38	79	24	18	26	425
k__Bacteria; p__Firmicutes; c__Clostridia; o__Clostridiales; f__Ruminococcaceae; g__Ruminococcaceae_UCG-009;	3	146	1	3	6	1	79	2
k__Bacteria; p__Firmicutes; c__Clostridia; o__Clostridiales; f__Ruminococcaceae; g__Ruminococcaceae_UCG-010;	102	86	6	128	1	0	266	3
k__Bacteria; p__Firmicutes; c__Clostridia; o__Clostridiales; f__Ruminococcaceae; g__Ruminococcaceae_UCG-013;	269	397	398	64	20	147	216	371
k__Bacteria; p__Firmicutes; c__Clostridia; o__Clostridiales; f__Ruminococcaceae; g__Ruminococcaceae_UCG-014;	299	1117	320	67	174	457	232	430
k__Bacteria; p__Firmicutes; c__Clostridia; o__Clostridiales; f__Ruminococcaceae; g__Ruminococcus_1;	377	154	440	449	72	462	209	113
k__Bacteria; p__Firmicutes; c__Clostridia; o__Clostridiales; f__Ruminococcaceae; g__Ruminococcus_2;	173	239	584	33	874	447	763	323
k__Bacteria; p__Firmicutes; c__Clostridia; o__Clostridiales; f__Ruminococcaceae; g__Saccharofermentans;	106	1	237	0	24	186	0	1
k__Bacteria; p__Firmicutes; c__Clostridia; o__Clostridiales; f__Ruminococcaceae; g__Subdoligranulum;	148	115	145	292	111	281	257	296

Taxonomy	OTUs							
	SM	PA	KD	LU	SP	LV	HE	BR
k__Bacteria; p__Firmicutes; c__Clostridia; o__Clostridiales; f__Ruminococcaceae; g__[Eubacterium]_coprostanoligenes_group;	910	358	581	242	269	732	380	1120
k__Bacteria; p__Firmicutes; c__Clostridia; o__Clostridiales; f__Ruminococcaceae; g__uncultured_bacterium_f_Ruminococcaceae;	3794	1623	1097	1330	1225	1032	1522	1393
k__Bacteria; p__Firmicutes; c__Clostridia; o__Clostridiales; f__uncultured_bacterium_o_Clostridiales; g__uncultured_bacterium_o_Clostridiales;	210	27	1	2	1	6	3	52
k__Bacteria; p__Firmicutes; c__Clostridia; o__DTU014; f__uncultured_bacterium_o_DTU014; g__uncultured_bacterium_o_DTU014;	88	83	68	13	13	8	16	8
k__Bacteria; p__Firmicutes; c__Clostridia; o__MBA03; f__uncultured_bacterium_o_MBA03; g__uncultured_bacterium_o_MBA03;	0	0	81	2	0	124	0	126
k__Bacteria; p__Firmicutes; c__Erysipelotrichia; o__Erysipelotrichales; f__Erysipelotrichaceae; g__Allobaculum;	183	207	338	110	157	293	206	339
k__Bacteria; p__Firmicutes; c__Erysipelotrichia; o__Erysipelotrichales; f__Erysipelotrichaceae; g__Candidatus_Stoquefichus;	0	1	38	0	0	1	0	3
k__Bacteria; p__Firmicutes; c__Erysipelotrichia; o__Erysipelotrichales; f__Erysipelotrichaceae; g__Dubosiella;	436	294	140	333	244	232	321	729
k__Bacteria; p__Firmicutes; c__Erysipelotrichia; o__Erysipelotrichales; f__Erysipelotrichaceae; g__Erysipelotrichaceae_UCG-003;	156	34	155	9	9	90	76	141
k__Bacteria; p__Firmicutes; c__Erysipelotrichia; o__Erysipelotrichales; f__Erysipelotrichaceae; g__Faecalibaculum;	206	2813	42	265	598	146	47	335
k__Bacteria; p__Firmicutes; c__Erysipelotrichia; o__Erysipelotrichales; f__Erysipelotrichaceae; g__Faecalitalea;	35	55	8	15	1	0	1	0
k__Bacteria; p__Firmicutes; c__Erysipelotrichia; o__Erysipelotrichales; f__Erysipelotrichaceae; g__Holdemanella;	14	13	16	20	17	265	179	6
k__Bacteria; p__Firmicutes; c__Erysipelotrichia; o__Erysipelotrichales; f__Erysipelotrichaceae; g__Ileibacterium;	37	1802	11	153	50	30	18	8
k__Bacteria; p__Firmicutes; c__Erysipelotrichia; o__Erysipelotrichales; f__Erysipelotrichaceae; g__Turicibacter;	761	1988	145	289	173	237	733	416
k__Bacteria; p__Firmicutes; c__Erysipelotrichia; o__Erysipelotrichales; f__Erysipelotrichaceae; g__[Clostridium]_innocuum_group;	16	0	58	1	0	9	64	43
k__Bacteria; p__Firmicutes; c__Erysipelotrichia; o__Erysipelotrichales; f__Erysipelotrichaceae; g__uncultured_bacterium_f_Erysipelotrichaceae;	28	15	12	5	26	10	51	42
k__Bacteria; p__Firmicutes; c__Negativicutes; o__Selenomonadales; f__Acidaminococcaceae; g__Phascolarctobacterium;	9	575	583	650	111	455	148	179
k__Bacteria; p__Firmicutes; c__Negativicutes; o__Selenomonadales; f__Acidaminococcaceae; g__Succiniclasticum;	130	211	325	90	184	518	520	0
k__Bacteria; p__Firmicutes; c__Negativicutes; o__Selenomonadales; f__Veillonellaceae; g__Dialister;	437	239	429	322	37	578	61	87
k__Bacteria; p__Firmicutes; c__Negativicutes; o__Selenomonadales; f__Veillonellaceae; g__Megamonas;	238	329	395	24	279	547	146	34
k__Bacteria; p__Firmicutes; c__Negativicutes; o__Selenomonadales; f__Veillonellaceae; g__Megasphaera;	132	156	660	213	200	351	239	150
k__Bacteria; p__Firmicutes; c__Negativicutes; o__Selenomonadales; f__Veillonellaceae; g__Mitsuokella;	8	74	76	16	214	287	9	9
k__Bacteria; p__Firmicutes; c__Negativicutes; o__Selenomonadales; f__Veillonellaceae; g__Pectinatus;	60	0	255	0	0	0	0	1
k__Bacteria; p__Firmicutes; c__Negativicutes; o__Selenomonadales; f__Veillonellaceae; g__Veillonella;	990	286	1015	245	789	1419	1272	723

Taxonomy	OTUs							
	SM	PA	KD	LU	SP	LV	HE	BR
k__Bacteria; p__Firmicutes; c__Negativicutes; o__Selenomonadales; f__Veillonellaceae; g__uncultured_bacterium_f_Veillonellaceae;	247	103	8	0	308	2	29	28
k__Bacteria; p__Firmicutes; c__uncultured_bacterium_p_Firmicutes; o__uncultured_bacterium_p_Firmicutes; f__uncultured_bacterium_p_Firmicutes; g__uncultured_bacterium_p_Firmicutes;	2	20	0	0	0	36	1	203
k__Bacteria; p__Fusobacteria; c__Fusobacteriia; o__Fusobacteriales; f__Fusobacteriaceae; g__Cetobacterium;	770	132	392	546	572	640	832	817
k__Bacteria; p__Fusobacteria; c__Fusobacteriia; o__Fusobacteriales; f__Fusobacteriaceae; g__Fusobacterium;	114	726	244	113	686	918	166	338
k__Bacteria; p__Fusobacteria; c__Fusobacteriia; o__Fusobacteriales; f__Fusobacteriaceae; g__Propionigenium;	2	1	0	0	0	33	242	2
k__Bacteria; p__Fusobacteria; c__Fusobacteriia; o__Fusobacteriales; f__Leptotrichiaceae; g__Leptotrichia;	55	1	0	2	0	200	26	30
k__Bacteria; p__Gemmatimonadetes; c__BD2-11_terrestrial_group; o__uncultured_bacterium_c_BD2-11_terrestrial_group; f__uncultured_bacterium_c_BD2-11_terrestrial_group; g__uncultured_bacterium_c_BD2-11_terrestrial_group;	329	3	0	2	0	0	0	0
k__Bacteria; p__Gemmatimonadetes; c__Gemmatimonadetes; o__Gemmatimonadales; f__Gemmatimonadaceae; g__Gemmatimonas;	98	236	859	340	1768	780	733	845
k__Bacteria; p__Gemmatimonadetes; c__Gemmatimonadetes; o__Gemmatimonadales; f__Gemmatimonadaceae; g__Gemmatirosa;	75	40	205	231	335	278	137	206
k__Bacteria; p__Gemmatimonadetes; c__Gemmatimonadetes; o__Gemmatimonadales; f__Gemmatimonadaceae; g__uncultured_bacterium_f_Gemmatimonadaceae;	2853	1183	3080	2912	2609	3764	1765	2149
k__Bacteria; p__Gemmatimonadetes; c__Longimicrobia; o__Longimicrobiales; f__Longimicrobiaceae; g__uncultured_bacterium_f_Longimicrobiaceae;	2	4	45	59	203	23	4	21
k__Bacteria; p__Gemmatimonadetes; c__S0134_terrestrial_group; o__uncultured_bacterium_c_S0134_terrestrial_group; f__uncultured_bacterium_c_S0134_terrestrial_group; g__uncultured_bacterium_c_S0134_terrestrial_group;	7	162	2	2	2	240	87	98
k__Bacteria; p__Nitrospirae; c__Nitrospira; o__Nitrospirales; f__Nitrospiraceae; g__Nitrospira;	303	99	164	175	12	84	294	155
k__Bacteria; p__Nitrospirae; c__Thermodesulfovibrionia; o__uncultured_bacterium_c_Thermodesulfovibrionia; f__uncultured_bacterium_c_Thermodesulfovibrionia; g__uncultured_bacterium_c_Thermodesulfovibrionia;	4	0	162	220	85	10	2	23
k__Bacteria; p__Patescibacteria; c__Berkelbacteria; o__uncultured_bacterium_c_Berkelbacteria; f__uncultured_bacterium_c_Berkelbacteria; g__uncultured_bacterium_c_Berkelbacteria;	0	0	0	0	0	0	1	316
k__Bacteria; p__Patescibacteria; c__Microgenomatia; o__Candidatus_Levybacteria; f__uncultured_bacterium_o_Candidatus_Levybacteria; g__uncultured_bacterium_o_Candidatus_Levybacteria;	0	0	0	1	0	0	362	1
k__Bacteria; p__Patescibacteria; c__Saccharimonadia; o__Saccharimonadales; f__Saccharimonadaceae; g__Candidatus_Saccharimonas;	126	931	51	24	11	21	67	121
k__Bacteria; p__Patescibacteria; c__Saccharimonadia; o__Saccharimonadales; f__Saccharimonadaceae; g__uncultured_bacterium_f_Saccharimonadaceae;	94	166	194	389	66	128	425	111
k__Bacteria; p__Patescibacteria; c__Saccharimonadia; o__Saccharimonadales; f__uncultured_bacterium_o_Saccharimonadales; g__uncultured_bacterium_o_Saccharimonadales;	677	24	414	232	475	444	84	381
k__Bacteria; p__Patescibacteria; c__uncultured_bacterium_p_Patescibacteria; o__uncultured_bacterium_p_Patescibacteria; f__uncultured_bacterium_p_Patescibacteria;	0	0	319	0	0	0	0	0

Taxonomy	OTUs							
	SM	PA	KD	LU	SP	LV	HE	BR
g__uncultured_bacterium_p_Patescibacteria;								
k__Bacteria; p__Planctomycetes; c__Pla4_lineage; o__uncultured_bacterium_c_Pla4_lineage; f__uncultured_bacterium_c_Pla4_lineage; g__uncultured_bacterium_c_Pla4_lineage;	0	87	138	8	1	0	0	3
k__Bacteria; p__Proteobacteria; c__Alphaproteobacteria; o__Acetobacterales; f__Acetobacteraceae; g__Acetobacter;	26	139	73	43	402	79	100	55
k__Bacteria; p__Proteobacteria; c__Alphaproteobacteria; o__Acetobacterales; f__Acetobacteraceae; g__Acidicaldus;	49	108	161	359	137	22	23	24
k__Bacteria; p__Proteobacteria; c__Alphaproteobacteria; o__Acetobacterales; f__Acetobacteraceae; g__Asaia;	0	0	2	160	3	237	0	2
k__Bacteria; p__Proteobacteria; c__Alphaproteobacteria; o__Acetobacterales; f__Acetobacteraceae; g__Craurococcus;	184	107	0	157	1	25	5	26
k__Bacteria; p__Proteobacteria; c__Alphaproteobacteria; o__Acetobacterales; f__Acetobacteraceae; g__Rhodovarius;	95	0	76	0	1	294	50	26
k__Bacteria; p__Proteobacteria; c__Alphaproteobacteria; o__Acetobacterales; f__Acetobacteraceae; g__Roseococcus;	586	257	340	430	64	35	620	29
k__Bacteria; p__Proteobacteria; c__Alphaproteobacteria; o__Acetobacterales; f__Acetobacteraceae; g__Roseomonas;	3	49	0	166	6	2	1	12
k__Bacteria; p__Proteobacteria; c__Alphaproteobacteria; o__Acetobacterales; f__Acetobacteraceae; g__Rubritepida;	1104	3	3	4	6	363	202	61
k__Bacteria; p__Proteobacteria; c__Alphaproteobacteria; o__Acetobacterales; f__Acetobacteraceae; g__uncultured_bacterium_f_Acetobacteraceae;	133	69	561	2237	470	272	443	687
k__Bacteria; p__Proteobacteria; c__Alphaproteobacteria; o__Azospirillales; f__Azospirillaceae; g__Azospirillum;	2	5	255	1	55	60	92	524
k__Bacteria; p__Proteobacteria; c__Alphaproteobacteria; o__Azospirillales; f__Azospirillaceae; g__Niveispirillum;	193	3	1	130	19	48	1	1
k__Bacteria; p__Proteobacteria; c__Alphaproteobacteria; o__Azospirillales; f__Azospirillaceae; g__Skermanella;	149	1	16	948	56	32	5	107
k__Bacteria; p__Proteobacteria; c__Alphaproteobacteria; o__Azospirillales; f__uncultured_bacterium_o_Azospirillales; g__uncultured_bacterium_o_Azospirillales;	69	49	478	171	273	426	99	69
k__Bacteria; p__Proteobacteria; c__Alphaproteobacteria; o__Caulobacterales; f__Caulobacteraceae; g__Brevundimonas;	743	566	633	597	666	1521	667	1556
k__Bacteria; p__Proteobacteria; c__Alphaproteobacteria; o__Caulobacterales; f__Caulobacteraceae; g__Caulobacter;	323	358	235	32	21	86	178	395
k__Bacteria; p__Proteobacteria; c__Alphaproteobacteria; o__Caulobacterales; f__Caulobacteraceae; g__Phenylobacterium;	331	245	196	679	18	441	69	280
k__Bacteria; p__Proteobacteria; c__Alphaproteobacteria; o__Caulobacterales; f__Caulobacteraceae; g__uncultured_bacterium_f_Caulobacteraceae;	609	365	824	126	655	191	268	67
k__Bacteria; p__Proteobacteria; c__Alphaproteobacteria; o__Caulobacterales; f__Hyphomonadaceae; g__Hirschia;	220	21	6	105	5	39	76	82
k__Bacteria; p__Proteobacteria; c__Alphaproteobacteria; o__Caulobacterales; f__Hyphomonadaceae; g__SWB02;	109	2	73	123	65	1	24	157
k__Bacteria; p__Proteobacteria; c__Alphaproteobacteria; o__Caulobacterales; f__Hyphomonadaceae; g__uncultured_bacterium_f_Hyphomonadaceae;	660	2759	477	2830	557	1187	1180	662
k__Bacteria; p__Proteobacteria; c__Alphaproteobacteria; o__Caulobacterales; f__Parvularculaceae; g__Amphiplicatus;	75	113	5	943	695	143	85	348
k__Bacteria; p__Proteobacteria; c__Alphaproteobacteria; o__Dongiales; f__Dongiaceae; g__Dongia;	64	245	134	352	77	246	401	1073

Taxonomy	OTUs							
	SM	PA	KD	LU	SP	LV	HE	BR
k__Bacteria; p__Proteobacteria; c__Alphaproteobacteria; o__Elsterales; f__URHD0088; g__uncultured_bacterium_f_URHD0088;	208	1	7	49	2	145	26	43
k__Bacteria; p__Proteobacteria; c__Alphaproteobacteria; o__Elsterales; f__uncultured_bacterium_o_Elsterales; g__uncultured_bacterium_o_Elsterales;	313	302	452	47	855	516	487	347
k__Bacteria; p__Proteobacteria; c__Alphaproteobacteria; o__Micropepsales; f__Micropepsaceae; g__uncultured_bacterium_f_Micropepsaceae;	84	358	86	446	255	521	357	494
k__Bacteria; p__Proteobacteria; c__Alphaproteobacteria; o__Paracaedibacterales; f__Paracaedibacteraceae; g__uncultured_bacterium_f_Paracaedibacteraceae;	38	0	9	116	1	60	3	12
k__Bacteria; p__Proteobacteria; c__Alphaproteobacteria; o__Parvibaculales; f__Parvibaculaceae; g__Parvibaculum;	26	2	0	287	4	134	2	206
k__Bacteria; p__Proteobacteria; c__Alphaproteobacteria; o__Puniceispirillales; f__EF100-94H03; g__uncultured_bacterium_f_EF100-94H03;	1	1	21	3	475	1	0	0
k__Bacteria; p__Proteobacteria; c__Alphaproteobacteria; o__Puniceispirillales; f__SAR116_clade; g__alpha_proteobacterium_SCGC_AAA015-N04;	0	1	0	0	1	47	173	1
k__Bacteria; p__Proteobacteria; c__Alphaproteobacteria; o__Reyranellales; f__Reyranellaceae; g__Reyranella;	196	180	324	288	108	702	517	328
k__Bacteria; p__Proteobacteria; c__Alphaproteobacteria; o__Reyranellales; f__Reyranellaceae; g__uncultured_bacterium_f_Reyranellaceae;	0	0	0	5	80	1	153	0
k__Bacteria; p__Proteobacteria; c__Alphaproteobacteria; o__Rhizobiales; f__A0839; g__uncultured_bacterium_f_A0839;	1	5	4	205	4	67	44	25
k__Bacteria; p__Proteobacteria; c__Alphaproteobacteria; o__Rhizobiales; f__Beijerinckiaceae; g__Bosea;	281	132	118	229	181	142	18	205
k__Bacteria; p__Proteobacteria; c__Alphaproteobacteria; o__Rhizobiales; f__Beijerinckiaceae; g__Methylobacterium;	685	435	481	395	384	1160	1582	406
k__Bacteria; p__Proteobacteria; c__Alphaproteobacteria; o__Rhizobiales; f__Beijerinckiaceae; g__Microvirga;	2	2	130	71	2	0	9	144
k__Bacteria; p__Proteobacteria; c__Alphaproteobacteria; o__Rhizobiales; f__Beijerinckiaceae; g__Psychroglaciecola;	3	239	0	1	1	0	1	1
k__Bacteria; p__Proteobacteria; c__Alphaproteobacteria; o__Rhizobiales; f__Beijerinckiaceae; g__Roseiarcus;	47	3	37	191	4	24	169	273
k__Bacteria; p__Proteobacteria; c__Alphaproteobacteria; o__Rhizobiales; f__Beijerinckiaceae; g__uncultured_bacterium_f_Beijerinckiaceae;	18	4571	13	21	10	183	263	121
k__Bacteria; p__Proteobacteria; c__Alphaproteobacteria; o__Rhizobiales; f__Devosiaceae; g__Devosia;	88	194	200	277	634	552	380	669
k__Bacteria; p__Proteobacteria; c__Alphaproteobacteria; o__Rhizobiales; f__Hyphomicrobiaceae; g__Hyphomicrobium;	366	200	186	357	64	241	651	355
k__Bacteria; p__Proteobacteria; c__Alphaproteobacteria; o__Rhizobiales; f__Hyphomicrobiaceae; g__Pedomicrobium;	36	171	274	557	78	40	344	73
k__Bacteria; p__Proteobacteria; c__Alphaproteobacteria; o__Rhizobiales; f__Hyphomicrobiaceae; g__uncultured_bacterium_f_Hyphomicrobiaceae;	121	86	93	742	8	200	64	214
k__Bacteria; p__Proteobacteria; c__Alphaproteobacteria; o__Rhizobiales; f__KF-JG30-B3; g__uncultured_bacterium_f_KF-JG30-B3;	26	151	2	43	1	128	54	21
k__Bacteria; p__Proteobacteria; c__Alphaproteobacteria; o__Rhizobiales; f__Methyloligellaceae; g__uncultured_bacterium_f_Methyloligellaceae;	160	37	188	233	96	209	27	164
k__Bacteria; p__Proteobacteria; c__Alphaproteobacteria; o__Rhizobiales; f__Methylopilaceae; g__uncultured_bacterium_f_Methylopilaceae;	0	0	261	2	0	40	0	1
k__Bacteria; p__Proteobacteria; c__Alphaproteobacteria; o__Rhizobiales; f__Pleomorphomonadaceae; g__Pleomorphomonas;	88	0	65	0	38	17	113	2

Taxonomy	OTUs							
	SM	PA	KD	LU	SP	LV	HE	BR
k__Bacteria; p__Proteobacteria; c__Alphaproteobacteria; o__Rhizobiales; f__Rhizobiaceae; g__Allorhizobium-Neorhizobium-Pararhizobium-Rhizobium;	407	529	484	419	1010	453	355	581
k__Bacteria; p__Proteobacteria; c__Alphaproteobacteria; o__Rhizobiales; f__Rhizobiaceae; g__Chelativorans;	51	0	1	93	120	0	64	2
k__Bacteria; p__Proteobacteria; c__Alphaproteobacteria; o__Rhizobiales; f__Rhizobiaceae; g__Mesorhizobium;	731	486	403	1340	966	248	1102	1336
*****k__Bacteria; p__Proteobacteria; c__Alphaproteobacteria; o__Rhizobiales; f__Rhizobiaceae; g__Ochrobactrum;	6142	4781	6871	7199	8165	3923	8266	6488
k__Bacteria; p__Proteobacteria; c__Alphaproteobacteria; o__Rhizobiales; f__Rhizobiaceae; g__uncultured_bacterium_f_Rhizobiaceae;	167	70	327	297	171	395	289	148
k__Bacteria; p__Proteobacteria; c__Alphaproteobacteria; o__Rhizobiales; f__Rhizobiales_Incertae_Sedis; g__Bauldia;	215	3	137	242	2	67	1	10
k__Bacteria; p__Proteobacteria; c__Alphaproteobacteria; o__Rhizobiales; f__Rhizobiales_Incertae_Sedis; g__Nordella;	174	298	7	164	4	101	86	262
k__Bacteria; p__Proteobacteria; c__Alphaproteobacteria; o__Rhizobiales; f__Rhizobiales_Incertae_Sedis; g__Phreatobacter;	3668	10515	4228	10007	4336	4469	3402	2566
k__Bacteria; p__Proteobacteria; c__Alphaproteobacteria; o__Rhizobiales; f__Rhizobiales_Incertae_Sedis; g__uncultured_bacterium_f_Rhizobiales_Incertae_Sedis;	2	22	334	33	1	84	28	140
k__Bacteria; p__Proteobacteria; c__Alphaproteobacteria; o__Rhizobiales; f__Stappiaceae; g__uncultured_bacterium_f_Stappiaceae;	5	0	99	10	6	76	2	50
k__Bacteria; p__Proteobacteria; c__Alphaproteobacteria; o__Rhizobiales; f__Xanthobacteraceae; g__Bradyrhizobium;	754	1000	1007	953	787	1073	1050	1093
k__Bacteria; p__Proteobacteria; c__Alphaproteobacteria; o__Rhizobiales; f__Xanthobacteraceae; g__Pseudolabrys;	826	803	772	304	94	1057	225	419
k__Bacteria; p__Proteobacteria; c__Alphaproteobacteria; o__Rhizobiales; f__Xanthobacteraceae; g__Rhodopseudomonas;	15	49	0	41	0	45	0	30
k__Bacteria; p__Proteobacteria; c__Alphaproteobacteria; o__Rhizobiales; f__Xanthobacteraceae; g__uncultured_bacterium_f_Xanthobacteraceae;	2519	1450	1305	2501	2075	1861	2304	1452
k__Bacteria; p__Proteobacteria; c__Alphaproteobacteria; o__Rhizobiales; f__uncultured_bacterium_o_Rhizobiales; g__uncultured_bacterium_o_Rhizobiales;	33	1	0	118	1	291	90	7
k__Bacteria; p__Proteobacteria; c__Alphaproteobacteria; o__Rhodobacterales; f__Rhodobacteraceae; g__Amaricoccus;	0	0	0	223	112	44	221	40
k__Bacteria; p__Proteobacteria; c__Alphaproteobacteria; o__Rhodobacterales; f__Rhodobacteraceae; g__Ascidiaceihabitans;	2	131	40	1	28	101	110	1
k__Bacteria; p__Proteobacteria; c__Alphaproteobacteria; o__Rhodobacterales; f__Rhodobacteraceae; g__Gemmobacter;	73	0	90	62	1	15	25	71
k__Bacteria; p__Proteobacteria; c__Alphaproteobacteria; o__Rhodobacterales; f__Rhodobacteraceae; g__HIMB11;	278	302	251	118	208	102	11	4
k__Bacteria; p__Proteobacteria; c__Alphaproteobacteria; o__Rhodobacterales; f__Rhodobacteraceae; g__Lentibacter;	0	0	79	4	57	57	1	4
k__Bacteria; p__Proteobacteria; c__Alphaproteobacteria; o__Rhodobacterales; f__Rhodobacteraceae; g__Paracoccus;	199	206	526	493	225	1078	299	166
k__Bacteria; p__Proteobacteria; c__Alphaproteobacteria; o__Rhodobacterales; f__Rhodobacteraceae; g__Rhodobacter;	173	539	94	62	156	101	17	32
k__Bacteria; p__Proteobacteria; c__Alphaproteobacteria; o__Rhodobacterales; f__Rhodobacteraceae; g__Roseobacter_clade_CHAB-I-5_lineage;	344	2	0	0	86	0	0	0

Taxonomy	OTUs							
	SM	PA	KD	LU	SP	LV	HE	BR
k__Bacteria; p__Proteobacteria; c__Alphaproteobacteria; o__Rhodobacterales; f__Rhodobacteraceae; g__Rubellimicrobium;	66	558	468	95	432	508	50	244
k__Bacteria; p__Proteobacteria; c__Alphaproteobacteria; o__Rhodobacterales; f__Rhodobacteraceae; g__uncultured_bacterium_f_Rhodobacteraceae;	805	54	162	664	1296	425	77	91
k__Bacteria; p__Proteobacteria; c__Alphaproteobacteria; o__Rhodospirillales; f__Rhodopirillaceae; g__Defluviicoccus;	288	0	0	698	1	75	1	19
k__Bacteria; p__Proteobacteria; c__Alphaproteobacteria; o__Rhodospirillales; f__Rhodospirillaceae; g__uncultured_bacterium_f_Rhodospirillaceae;	4	35	3	3	1	156	0	239
k__Bacteria; p__Proteobacteria; c__Alphaproteobacteria; o__Rhodospirillales; f__uncultured_bacterium_o_Rhodospirillales; g__uncultured_bacterium_o_Rhodospirillales;	62	28	2	0	244	29	2	433
k__Bacteria; p__Proteobacteria; c__Alphaproteobacteria; o__Rickettsiales; f__Mitochondria; g__uncultured_bacterium_f_Mitochondria;	540	1139	1615	906	1273	1299	2287	522
k__Bacteria; p__Proteobacteria; c__Alphaproteobacteria; o__Rickettsiales; f__Rickettsiaceae; g__Candidatus_Megaira;	1	291	0	9	1	0	0	0
k__Bacteria; p__Proteobacteria; c__Alphaproteobacteria; o__Rickettsiales; f__SM2D12; g__uncultured_bacterium_f_SM2D12;	0	0	0	1	1	0	424	0
k__Bacteria; p__Proteobacteria; c__Alphaproteobacteria; o__SAR11_clade; f__Clade_I; g__uncultured_bacterium_f_Clade_I;	0	155	18	2	164	61	0	13
k__Bacteria; p__Proteobacteria; c__Alphaproteobacteria; o__Sphingomonadales; f__Sphingomonadaceae; g__Altererythrobacter;	445	20	380	249	404	351	117	322
k__Bacteria; p__Proteobacteria; c__Alphaproteobacteria; o__Sphingomonadales; f__Sphingomonadaceae; g__Croceicoccus;	33	17	5	21	70	244	49	46
k__Bacteria; p__Proteobacteria; c__Alphaproteobacteria; o__Sphingomonadales; f__Sphingomonadaceae; g__DSSF69;	532	11	5	1002	138	264	71	72
k__Bacteria; p__Proteobacteria; c__Alphaproteobacteria; o__Sphingomonadales; f__Sphingomonadaceae; g__Erythrobacter;	232	74	353	355	521	187	149	506
k__Bacteria; p__Proteobacteria; c__Alphaproteobacteria; o__Sphingomonadales; f__Sphingomonadaceae; g__Novosphingobium;	524	466	903	936	470	1234	725	602
k__Bacteria; p__Proteobacteria; c__Alphaproteobacteria; o__Sphingomonadales; f__Sphingomonadaceae; g__Porphyrobacter;	925	1325	318	3379	238	854	283	589
k__Bacteria; p__Proteobacteria; c__Alphaproteobacteria; o__Sphingomonadales; f__Sphingomonadaceae; g__Sphingobium;	471	91	112	12	61	316	123	118
*****k__Bacteria; p__Proteobacteria; c__Alphaproteobacteria; o__Sphingomonadales; f__Sphingomonadaceae; g__Sphingomonas;	7266	8538	11520	5433	7868	16005	8460	6829
k__Bacteria; p__Proteobacteria; c__Alphaproteobacteria; o__Sphingomonadales; f__Sphingomonadaceae; g__Sphingopyxis;	405	322	164	106	10	503	153	194
k__Bacteria; p__Proteobacteria; c__Alphaproteobacteria; o__Sphingomonadales; f__Sphingomonadaceae; g__uncultured_bacterium_f_Sphingomonadaceae;	383	789	509	387	433	2185	1196	577
k__Bacteria; p__Proteobacteria; c__Alphaproteobacteria; o__Tistrellales; f__Geminicoccaceae; g__Candidatus_Alysiosphaera;	1	0	302	18	0	90	0	5
k__Bacteria; p__Proteobacteria; c__Alphaproteobacteria; o__Tistrellales; f__Geminicoccaceae; g__uncultured_bacterium_f_Geminicoccaceae;	42	72	4	1785	39	163	97	417
k__Bacteria; p__Proteobacteria; c__Alphaproteobacteria; o__uncultured_bacterium_c_Alphaproteobacteria; f__uncultured_bacterium_c_Alphaproteobacteria; g__uncultured_bacterium_c_Alphaproteobacteria;	1389	320	134	886	695	771	1095	260

Taxonomy	OTUs							
	SM	PA	KD	LU	SP	LV	HE	BR
k__Bacteria; p__Proteobacteria; c__Deltaproteobacteria; o__Bdellovibrionales; f__Bdellovibrionaceae; g__Bdellovibrio;	29	140	20	90	42	97	273	27
k__Bacteria; p__Proteobacteria; c__Deltaproteobacteria; o__Bdellovibrionales; f__Bdellovibrionaceae; g__OM27_clade;	4	0	356	0	0	0	0	2
k__Bacteria; p__Proteobacteria; c__Deltaproteobacteria; o__Deltaproteobacteria_Incertae_Sedis; f__Syntrophorhabdaceae; g__Syntrophorhabdus;	327	4	2	567	49	6	3	13
k__Bacteria; p__Proteobacteria; c__Deltaproteobacteria; o__Desulfarculales; f__Desulfarculaceae; g__uncultured_bacterium_f_Desulfarculaceae;	104	225	2	165	445	366	380	65
k__Bacteria; p__Proteobacteria; c__Deltaproteobacteria; o__Desulfobacterales; f__Desulfobacteraceae; g__Desulfococcus;	4	141	1	11	41	6	66	1
k__Bacteria; p__Proteobacteria; c__Deltaproteobacteria; o__Desulfobacterales; f__Desulfobulbaceae; g__uncultured_bacterium_f_Desulfobulbaceae;	0	0	291	0	0	1	1	0
k__Bacteria; p__Proteobacteria; c__Deltaproteobacteria; o__Desulfovibrionales; f__Desulfomicrobiaceae; g__Desulfomicrobium;	0	0	4	0	280	1	0	0
k__Bacteria; p__Proteobacteria; c__Deltaproteobacteria; o__Desulfovibrionales; f__Desulfovibrionaceae; g__Desulfovibrio;	2208	3313	386	843	147	326	424	526
k__Bacteria; p__Proteobacteria; c__Deltaproteobacteria; o__Desulfovibrionales; f__Desulfovibrionaceae; g__uncultured_bacterium_f_Desulfovibrionaceae;	138	192	10	229	9	135	148	760
k__Bacteria; p__Proteobacteria; c__Deltaproteobacteria; o__Myxococcales; f__BIrii41; g__uncultured_bacterium_f_BIrii41;	0	7	3	4	2	318	36	214
k__Bacteria; p__Proteobacteria; c__Deltaproteobacteria; o__Myxococcales; f__Eel-36e1D6; g__uncultured_bacterium_f_Eel-36e1D6;	309	2	0	1	0	0	0	1
k__Bacteria; p__Proteobacteria; c__Deltaproteobacteria; o__Myxococcales; f__Haliangiaceae; g__Haliangium;	298	279	866	1189	558	677	270	638
k__Bacteria; p__Proteobacteria; c__Deltaproteobacteria; o__Myxococcales; f__P3OB-42; g__uncultured_bacterium_f_P3OB-42;	1	0	0	0	0	0	269	1
k__Bacteria; p__Proteobacteria; c__Deltaproteobacteria; o__Myxococcales; f__Polyangiaceae; g__Pajaroellobacter;	47	177	567	16	922	252	727	859
k__Bacteria; p__Proteobacteria; c__Deltaproteobacteria; o__Myxococcales; f__Polyangiaceae; g__Polyangium;	0	2	54	0	0	0	607	11
k__Bacteria; p__Proteobacteria; c__Deltaproteobacteria; o__Myxococcales; f__Polyangiaceae; g__uncultured_bacterium_f_Polyangiaceae;	0	1	125	22	1	42	0	62
k__Bacteria; p__Proteobacteria; c__Deltaproteobacteria; o__Myxococcales; f__Sandaracinaceae; g__uncultured_bacterium_f_Sandaracinaceae;	0	0	0	0	1	281	0	0
k__Bacteria; p__Proteobacteria; c__Deltaproteobacteria; o__Myxococcales; f__bacteriap25; g__uncultured_bacterium_f_bacteriap25;	41	67	535	324	3	109	84	251
k__Bacteria; p__Proteobacteria; c__Deltaproteobacteria; o__Myxococcales; f__uncultured_bacterium_o_Myxococcales; g__uncultured_bacterium_o_Myxococcales;	293	2	2	3	2	290	59	375
k__Bacteria; p__Proteobacteria; c__Deltaproteobacteria; o__NB1-j; f__uncultured_bacterium_o_NB1-j; g__uncultured_bacterium_o_NB1-j;	0	0	2	414	0	0	0	0
k__Bacteria; p__Proteobacteria; c__Deltaproteobacteria; o__Oligoflexales; f__0319-6G20; g__uncultured_bacterium_f_0319-6G20;	309	2	417	240	23	4	26	3
k__Bacteria; p__Proteobacteria; c__Deltaproteobacteria; o__Oligoflexales; f__Oligoflexaceae; g__uncultured_bacterium_f_Oligoflexaceae;	0	0	326	0	1	0	0	1
k__Bacteria; p__Proteobacteria; c__Deltaproteobacteria; o__SAR324_cladeMarine_group_B; f__bacterium_enrichment_culture_clone_B302011; g__uncultured_bacterium_f_bacterium_enrichment_culture_clone_B302011;	56	189	0	44	235	2	1	6

Taxonomy	OTUs							
	SM	PA	KD	LU	SP	LV	HE	BR
k__Bacteria; p__Proteobacteria; c__Deltaproteobacteria; o__Sva0485; f__uncultured_bacterium_o_Sva0485; g__uncultured_bacterium_o_Sva0485;	230	52	0	1	0	0	0	13
k__Bacteria; p__Proteobacteria; c__Deltaproteobacteria; o__Syntrophobacterales; f__Syntrophaceae; g__Desulfobacca;	1	0	0	280	0	0	0	0
k__Bacteria; p__Proteobacteria; c__Deltaproteobacteria; o__Syntrophobacterales; f__Syntrophaceae; g__Smithella;	0	5	234	0	0	1	0	1
k__Bacteria; p__Proteobacteria; c__Deltaproteobacteria; o__Syntrophobacterales; f__Syntrophaceae; g__uncultured_bacterium_f_Syntrophaceae;	379	1	0	10	121	2	2	271
k__Bacteria; p__Proteobacteria; c__Deltaproteobacteria; o__uncultured_bacterium_c_Deltaproteobacteria; f__uncultured_bacterium_c_Deltaproteobacteria; g__uncultured_bacterium_c_Deltaproteobacteria;	475	49	357	589	786	806	569	505
k__Bacteria; p__Proteobacteria; c__Gammaproteobacteria; o__Aeromonadales; f__Aeromonadaceae; g__Aeromonas;	217	15	145	14	14	142	187	97
k__Bacteria; p__Proteobacteria; c__Gammaproteobacteria; o__Aeromonadales; f__Aeromonadaceae; g__Tolumonas;	1	34	232	4	180	0	120	0
k__Bacteria; p__Proteobacteria; c__Gammaproteobacteria; o__Aeromonadales; f__Succinivibrionaceae; g__Anaerobiospirillum;	18	0	13	0	60	28	188	0
k__Bacteria; p__Proteobacteria; c__Gammaproteobacteria; o__Aeromonadales; f__Succinivibrionaceae; g__Succinivibrio;	394	431	405	13	580	1221	323	1
k__Bacteria; p__Proteobacteria; c__Gammaproteobacteria; o__Alteromonadales; f__Alteromonadaceae; g__Rheinheimera;	10	2	7	64	394	0	2	196
k__Bacteria; p__Proteobacteria; c__Gammaproteobacteria; o__Alteromonadales; f__Idiomarinaceae; g__Aliidiomarina;	110	733	416	386	1164	179	189	331
k__Bacteria; p__Proteobacteria; c__Gammaproteobacteria; o__Alteromonadales; f__Idiomarinaceae; g__Idiomarina;	0	3	0	0	1	0	2	282
k__Bacteria; p__Proteobacteria; c__Gammaproteobacteria; o__Alteromonadales; f__Shewanellaceae; g__Shewanella;	1	2	87	87	3	147	2	5
k__Bacteria; p__Proteobacteria; c__Gammaproteobacteria; o__Betaproteobacteriales; f__B1-7BS; g__uncultured_bacterium_f_B1-7BS;	0	0	0	672	0	0	0	4
k__Bacteria; p__Proteobacteria; c__Gammaproteobacteria; o__Betaproteobacteriales; f__Burkholderiaceae; g__Achromobacter;	61	70	222	500	252	143	163	408
k__Bacteria; p__Proteobacteria; c__Gammaproteobacteria; o__Betaproteobacteriales; f__Burkholderiaceae; g__Acidovorax;	64	139	385	239	35	122	3	50
k__Bacteria; p__Proteobacteria; c__Gammaproteobacteria; o__Betaproteobacteriales; f__Burkholderiaceae; g__Aquabacterium;	30	129	769	232	311	54	110	320
k__Bacteria; p__Proteobacteria; c__Gammaproteobacteria; o__Betaproteobacteriales; f__Burkholderiaceae; g__Burkholderia-Caballeronia-Paraburkholderia;	631	1064	1496	538	639	737	280	640
k__Bacteria; p__Proteobacteria; c__Gammaproteobacteria; o__Betaproteobacteriales; f__Burkholderiaceae; g__Candidatus_Vidania;	7	10	16	36	65	10	93	27
k__Bacteria; p__Proteobacteria; c__Gammaproteobacteria; o__Betaproteobacteriales; f__Burkholderiaceae; g__Comamonas;	141	458	507	32	444	143	636	178
*****k__Bacteria; p__Proteobacteria; c__Gammaproteobacteria; o__Betaproteobacteriales; f__Burkholderiaceae; g__Cupriavidus;	3359<br>6	4048<br>1	5510<br>7	3506<br>0	4223<br>2	2081<br>6	5624<br>6	4731<br>5
k__Bacteria; p__Proteobacteria; c__Gammaproteobacteria; o__Betaproteobacteriales; f__Burkholderiaceae; g__Curvibacter;	8	113	301	111	61	650	573	165

Taxonomy	OTUs							
	SM	PA	KD	LU	SP	LV	HE	BR
k__Bacteria; p__Proteobacteria; c__Gammaproteobacteria; o__Betaproteobacteriales; f__Burkholderiaceae; g__Delftia;	2189	741	1931	1400	1629	1217	1395	1279
k__Bacteria; p__Proteobacteria; c__Gammaproteobacteria; o__Betaproteobacteriales; f__Burkholderiaceae; g__Hydrogenophaga;	692	407	1185	215	531	1366	1584	1573
k__Bacteria; p__Proteobacteria; c__Gammaproteobacteria; o__Betaproteobacteriales; f__Burkholderiaceae; g__Inhella;	88	114	128	5	101	99	45	47
k__Bacteria; p__Proteobacteria; c__Gammaproteobacteria; o__Betaproteobacteriales; f__Burkholderiaceae; g__Lautropia;	92	21	3	222	311	161	131	200
k__Bacteria; p__Proteobacteria; c__Gammaproteobacteria; o__Betaproteobacteriales; f__Burkholderiaceae; g__Limnobacter;	58	53	105	238	109	4098	2544	4127
k__Bacteria; p__Proteobacteria; c__Gammaproteobacteria; o__Betaproteobacteriales; f__Burkholderiaceae; g__Massilia;	117	582	583	298	1061	363	541	356
k__Bacteria; p__Proteobacteria; c__Gammaproteobacteria; o__Betaproteobacteriales; f__Burkholderiaceae; g__Noviherbaspirillum;	0	1	297	0	1	0	0	0
k__Bacteria; p__Proteobacteria; c__Gammaproteobacteria; o__Betaproteobacteriales; f__Burkholderiaceae; g__Ottowia;	5	0	1	51	1	149	54	36
k__Bacteria; p__Proteobacteria; c__Gammaproteobacteria; o__Betaproteobacteriales; f__Burkholderiaceae; g__Parasutterella;	577	396	624	1338	1129	341	336	373
k__Bacteria; p__Proteobacteria; c__Gammaproteobacteria; o__Betaproteobacteriales; f__Burkholderiaceae; g__Pelistega;	189	2	189	49	462	120	195	1
k__Bacteria; p__Proteobacteria; c__Gammaproteobacteria; o__Betaproteobacteriales; f__Burkholderiaceae; g__Pelomonas;	0	39	275	94	63	51	137	6
k__Bacteria; p__Proteobacteria; c__Gammaproteobacteria; o__Betaproteobacteriales; f__Burkholderiaceae; g__Polaromonas;	28	1	4	5	64	271	116	128
k__Bacteria; p__Proteobacteria; c__Gammaproteobacteria; o__Betaproteobacteriales; f__Burkholderiaceae; g__Pseudoduganella;	109	2	19	72	1	114	10	11
*****k__Bacteria; p__Proteobacteria; c__Gammaproteobacteria; o__Betaproteobacteriales; f__Burkholderiaceae; g__Ralstonia;	1609	1551	1403	1244	2153	1001	2133	1572
	6	2	5	8	1	5	9	3
k__Bacteria; p__Proteobacteria; c__Gammaproteobacteria; o__Betaproteobacteriales; f__Burkholderiaceae; g__Ramlibacter;	67	110	132	194	10	259	112	149
k__Bacteria; p__Proteobacteria; c__Gammaproteobacteria; o__Betaproteobacteriales; f__Burkholderiaceae; g__Rhizobacter;	24	21	107	5	77	199	147	94
k__Bacteria; p__Proteobacteria; c__Gammaproteobacteria; o__Betaproteobacteriales; f__Burkholderiaceae; g__Sutterella;	65	15	109	39	85	7	512	4
k__Bacteria; p__Proteobacteria; c__Gammaproteobacteria; o__Betaproteobacteriales; f__Burkholderiaceae; g__Tepidimonas;	100	0	87	60	48	25	0	3
k__Bacteria; p__Proteobacteria; c__Gammaproteobacteria; o__Betaproteobacteriales; f__Burkholderiaceae; g__uncultured_bacterium_f_Burkholderiaceae;	1986	698	3262	2494	2503	2989	1557	2605
k__Bacteria; p__Proteobacteria; c__Gammaproteobacteria; o__Betaproteobacteriales; f__Hydrogenophilaceae; g__Thiobacillus;	246	1	19	25	277	8	210	23
k__Bacteria; p__Proteobacteria; c__Gammaproteobacteria; o__Betaproteobacteriales; f__Methylophilaceae; g__Methylophilus;	59	133	270	16	16	712	390	950
k__Bacteria; p__Proteobacteria; c__Gammaproteobacteria; o__Betaproteobacteriales; f__Methylophilaceae; g__Methylotenera;	0	9	8	23	1	157	111	114
k__Bacteria; p__Proteobacteria; c__Gammaproteobacteria; o__Betaproteobacteriales; f__Neisseriaceae; g__Neisseria;	166	53	143	507	432	188	811	301

Taxonomy	OTUs							
	SM	PA	KD	LU	SP	LV	HE	BR
k__Bacteria; p__Proteobacteria; c__Gammaproteobacteria; o__Betaproteobacteriales; f__Neisseriaceae; g__uncultured_bacterium_f_Neisseriaceae;	70	64	62	42	7	6	2	123
k__Bacteria; p__Proteobacteria; c__Gammaproteobacteria; o__Betaproteobacteriales; f__Nitrosomonadaceae; g__DSSD61;	2	0	243	144	3	12	54	3
k__Bacteria; p__Proteobacteria; c__Gammaproteobacteria; o__Betaproteobacteriales; f__Nitrosomonadaceae; g__Ellin6067;	319	279	471	517	550	743	325	956
k__Bacteria; p__Proteobacteria; c__Gammaproteobacteria; o__Betaproteobacteriales; f__Nitrosomonadaceae; g__IS-44;	3	277	17	3	6	94	26	38
k__Bacteria; p__Proteobacteria; c__Gammaproteobacteria; o__Betaproteobacteriales; f__Nitrosomonadaceae; g__MND1;	979	1136	997	978	1079	1513	1424	1075
k__Bacteria; p__Proteobacteria; c__Gammaproteobacteria; o__Betaproteobacteriales; f__Nitrosomonadaceae; g__Nitrosospira;	104	2	170	35	0	88	56	65
k__Bacteria; p__Proteobacteria; c__Gammaproteobacteria; o__Betaproteobacteriales; f__Nitrosomonadaceae; g__mle1-7;	223	3	187	15	92	220	1	1
k__Bacteria; p__Proteobacteria; c__Gammaproteobacteria; o__Betaproteobacteriales; f__Rhodocyclaceae; g__Dechloromonas;	98	550	282	346	73	226	1117	89
k__Bacteria; p__Proteobacteria; c__Gammaproteobacteria; o__Betaproteobacteriales; f__Rhodocyclaceae; g__Methyloversatilis;	94	132	299	339	97	295	36	47
k__Bacteria; p__Proteobacteria; c__Gammaproteobacteria; o__Betaproteobacteriales; f__Rhodocyclaceae; g__Thauera;	348	150	288	770	198	398	286	242
k__Bacteria; p__Proteobacteria; c__Gammaproteobacteria; o__Betaproteobacteriales; f__Rhodocyclaceae; g__uncultured_bacterium_f_Rhodocyclaceae;	39	265	37	547	2	11	312	12
k__Bacteria; p__Proteobacteria; c__Gammaproteobacteria; o__Betaproteobacteriales; f__SC-I-84; g__beta_proteobacterium_JGI_0001003-N18;	64	1	56	2	0	133	44	2
k__Bacteria; p__Proteobacteria; c__Gammaproteobacteria; o__Betaproteobacteriales; f__SC-I-84; g__uncultured_bacterium_f_SC-I-84;	456	326	735	799	503	737	474	410
k__Bacteria; p__Proteobacteria; c__Gammaproteobacteria; o__Betaproteobacteriales; f__TRA3-20; g__uncultured_bacterium_f_TRA3-20;	642	60	297	573	336	600	662	205
k__Bacteria; p__Proteobacteria; c__Gammaproteobacteria; o__Betaproteobacteriales; f__uncultured_bacterium_o_Betaproteobacteriales; g__uncultured_bacterium_o_Betaproteobacteriales;	0	1	0	0	235	0	0	1
k__Bacteria; p__Proteobacteria; c__Gammaproteobacteria; o__CCD24; f__uncultured_bacterium_o_CCD24; g__uncultured_bacterium_o_CCD24;	246	28	135	213	489	524	62	263
k__Bacteria; p__Proteobacteria; c__Gammaproteobacteria; o__CCM19a; f__uncultured_bacterium_o_CCM19a; g__uncultured_bacterium_o_CCM19a;	32	0	0	148	0	56	33	0
k__Bacteria; p__Proteobacteria; c__Gammaproteobacteria; o__Cellvibrionales; f__Spongiibacteraceae; g__BD1-7_clade;	2	73	39	84	53	82	6	0
k__Bacteria; p__Proteobacteria; c__Gammaproteobacteria; o__Diplorickettsiales; f__Diplorickettsiaceae; g__Aquicella;	397	4	0	2	0	1	0	3
k__Bacteria; p__Proteobacteria; c__Gammaproteobacteria; o__Diplorickettsiales; f__Diplorickettsiaceae; g__uncultured_bacterium_f_Diplorickettsiaceae;	0	519	248	1	301	0	0	3
k__Bacteria; p__Proteobacteria; c__Gammaproteobacteria; o__Enterobacteriales; f__Enterobacteriaceae; g__Arsenophonus;	0	60	11	0	173	43	5	0
k__Bacteria; p__Proteobacteria; c__Gammaproteobacteria; o__Enterobacteriales; f__Enterobacteriaceae; g__Escherichia-Shigella;	1182	1114	1644	874	1160	2138	2065	1120
k__Bacteria; p__Proteobacteria; c__Gammaproteobacteria; o__Enterobacteriales; f__Enterobacteriaceae; g__Pantoea;	32	10	48	48	45	14	39	77
k__Bacteria; p__Proteobacteria; c__Gammaproteobacteria; o__Enterobacteriales; f__Enterobacteriaceae; g__Pectobacterium;	1	1	83	44	4	65	7	5

Taxonomy	OTUs							
	SM	PA	KD	LU	SP	LV	HE	BR
k__Bacteria; p__Proteobacteria; c__Gammaproteobacteria; o__Enterobacteriales; f__Enterobacteriaceae; g__Plesiomonas;	86	4	3	2	102	75	29	73
k__Bacteria; p__Proteobacteria; c__Gammaproteobacteria; o__Enterobacteriales; f__Enterobacteriaceae; g__Proteus;	13	459	9	443	10	18	25	49
k__Bacteria; p__Proteobacteria; c__Gammaproteobacteria; o__Enterobacteriales; f__Enterobacteriaceae; g__Rahnella;	1	214	0	1	3	53	0	151
k__Bacteria; p__Proteobacteria; c__Gammaproteobacteria; o__Enterobacteriales; f__Enterobacteriaceae; g__Serratia;	234	50	127	102	212	576	469	1049
*****k__Bacteria; p__Proteobacteria; c__Gammaproteobacteria; o__Enterobacteriales; f__Enterobacteriaceae; g__uncultured_bacterium_f_Enterobacteriaceae;	7282	5555	8388	7632	7743	7040	8237	6180
k__Bacteria; p__Proteobacteria; c__Gammaproteobacteria; o__Francisellales; f__Francisellaceae; g__Francisella;	0	40	0	1	133	58	102	0
k__Bacteria; p__Proteobacteria; c__Gammaproteobacteria; o__Gammaproteobacteria_Incertae_Sedis; f__uncultured_bacterium_o_Gammaproteobacteria_Incertae_Sedis; g__uncultured_bacterium_o_Gammaproteobacteria_Incertae_Sedis;	390	378	376	759	1050	1349	879	1080
k__Bacteria; p__Proteobacteria; c__Gammaproteobacteria; o__KF-JG30-C25; f__uncultured_bacterium_o_KF-JG30-C25; g__uncultured_bacterium_o_KF-JG30-C25;	133	181	129	46	2	349	126	10
k__Bacteria; p__Proteobacteria; c__Gammaproteobacteria; o__Methylococcales; f__Methylomonaceae; g__Methylomonas;	31	0	1	95	0	243	1	1
k__Bacteria; p__Proteobacteria; c__Gammaproteobacteria; o__Nitrococcales; f__Nitrococcales_Incertae_Sedis; g__Methylonatrum;	21	227	107	96	45	131	178	13
k__Bacteria; p__Proteobacteria; c__Gammaproteobacteria; o__Oceanospirillales; f__Halomonadaceae; g__Candidatus_Portiera;	0	0	109	0	0	3	1	166
*****k__Bacteria; p__Proteobacteria; c__Gammaproteobacteria; o__Oceanospirillales; f__Halomonadaceae; g__Halomonas;	7525	2828<br>2	2503<br>3	2831<br>2	4266<br>9	1504<br>7	2547<br>8	1215<br>7
k__Bacteria; p__Proteobacteria; c__Gammaproteobacteria; o__Oceanospirillales; f__Halomonadaceae; g__Salinicola;	0	251	0	1	0	0	0	0
k__Bacteria; p__Proteobacteria; c__Gammaproteobacteria; o__PLTA13; f__uncultured_bacterium_o_PLTA13; g__uncultured_bacterium_o_PLTA13;	300	156	186	1124	234	505	213	354
k__Bacteria; p__Proteobacteria; c__Gammaproteobacteria; o__Pasteurellales; f__Pasteurellaceae; g__Haemophilus;	141	37	28	35	66	1	0	36
k__Bacteria; p__Proteobacteria; c__Gammaproteobacteria; o__Pasteurellales; f__Pasteurellaceae; g__Rodentibacter;	93	1009	3	153	279	87	69	59
k__Bacteria; p__Proteobacteria; c__Gammaproteobacteria; o__Pseudomonadales; f__Moraxellaceae; g__Acinetobacter;	4060	3949	5340	3973	6852	6398	4334	7366
k__Bacteria; p__Proteobacteria; c__Gammaproteobacteria; o__Pseudomonadales; f__Moraxellaceae; g__Enhydrobacter;	412	1043	973	403	2337	1063	764	771
k__Bacteria; p__Proteobacteria; c__Gammaproteobacteria; o__Pseudomonadales; f__Moraxellaceae; g__Psychrobacter;	42	23	3	53	142	1	13	126
k__Bacteria; p__Proteobacteria; c__Gammaproteobacteria; o__Pseudomonadales; f__Pseudomonadaceae; g__Pseudomonas;	1987	1641	1539	1921	2674	2913	2544	1301
k__Bacteria; p__Proteobacteria; c__Gammaproteobacteria; o__Pseudomonadales; f__Pseudomonadaceae; g__uncultured_bacterium_f_Pseudomonadaceae;	56	32	69	1	7	42	227	1
k__Bacteria; p__Proteobacteria; c__Gammaproteobacteria; o__R7C24; f__uncultured_bacterium_o_R7C24; g__uncultured_bacterium_o_R7C24;	10	59	135	5	3	155	130	195

Taxonomy	OTUs							
	SM	PA	KD	LU	SP	LV	HE	BR
k__Bacteria; p__Proteobacteria; c__Gammaproteobacteria; o__SAR86_clade; f__uncultured_bacterium_o_SAR86_clade; g__uncultured_bacterium_o_SAR86_clade;	0	17	236	142	94	446	39	0
k__Bacteria; p__Proteobacteria; c__Gammaproteobacteria; o__SZB30; f__uncultured_bacterium_o_SZB30; g__uncultured_bacterium_o_SZB30;	1	39	285	116	98	65	176	14
k__Bacteria; p__Proteobacteria; c__Gammaproteobacteria; o__Salinisphaerales; f__Solimonadaceae; g__Nevskia;	2	5	352	595	1	84	210	65
k__Bacteria; p__Proteobacteria; c__Gammaproteobacteria; o__Salinisphaerales; f__Solimonadaceae; g__Polycyclovorans;	157	132	407	378	157	173	188	256
k__Bacteria; p__Proteobacteria; c__Gammaproteobacteria; o__Steroidobacterales; f__Steroidobacteraceae; g__Steroidobacter;	94	208	277	1840	292	503	140	87
k__Bacteria; p__Proteobacteria; c__Gammaproteobacteria; o__Steroidobacterales; f__Steroidobacteraceae; g__uncultured_bacterium_f_Steroidobacteraceae;	384	528	153	1102	309	606	75	131
k__Bacteria; p__Proteobacteria; c__Gammaproteobacteria; o__Steroidobacterales; f__Woeseiaceae; g__Woeseia;	256	0	2	2	0	0	1	2
k__Bacteria; p__Proteobacteria; c__Gammaproteobacteria; o__UBA10353_marine_group; f__uncultured_bacterium_o_UBA10353_marine_group; g__uncultured_bacterium_o_UBA10353_marine_group;	0	98	78	0	0	61	0	0
k__Bacteria; p__Proteobacteria; c__Gammaproteobacteria; o__Vibrionales; f__Vibrionaceae; g__Photobacterium;	2	108	210	1	2	153	1	1
k__Bacteria; p__Proteobacteria; c__Gammaproteobacteria; o__Vibrionales; f__Vibrionaceae; g__Vibrio;	249	524	722	698	166	612	163	175
k__Bacteria; p__Proteobacteria; c__Gammaproteobacteria; o__WD260; f__uncultured_bacterium_o_WD260; g__uncultured_bacterium_o_WD260;	1	0	76	318	0	29	0	0
k__Bacteria; p__Proteobacteria; c__Gammaproteobacteria; o__Xanthomonadales; f__Rhodanobacteraceae; g__Chujaibacter;	18	77	445	13	91	228	234	36
k__Bacteria; p__Proteobacteria; c__Gammaproteobacteria; o__Xanthomonadales; f__Rhodanobacteraceae; g__Dyella;	73	70	58	3	34	50	164	52
k__Bacteria; p__Proteobacteria; c__Gammaproteobacteria; o__Xanthomonadales; f__Rhodanobacteraceae; g__Rhodanobacter;	393	346	973	139	561	358	179	155
k__Bacteria; p__Proteobacteria; c__Gammaproteobacteria; o__Xanthomonadales; f__Rhodanobacteraceae; g__uncultured_bacterium_f_Rhodanobacteraceae;	74	7	245	131	48	141	405	532
k__Bacteria; p__Proteobacteria; c__Gammaproteobacteria; o__Xanthomonadales; f__Xanthomonadaceae; g__Arenimonas;	16	52	2	3	26	221	523	351
k__Bacteria; p__Proteobacteria; c__Gammaproteobacteria; o__Xanthomonadales; f__Xanthomonadaceae; g__Luteimonas;	86	117	65	55	279	418	417	62
k__Bacteria; p__Proteobacteria; c__Gammaproteobacteria; o__Xanthomonadales; f__Xanthomonadaceae; g__Lysobacter;	137	342	649	418	465	982	709	162
k__Bacteria; p__Proteobacteria; c__Gammaproteobacteria; o__Xanthomonadales; f__Xanthomonadaceae; g__Pseudoxanthomonas;	19	97	79	68	56	77	2	28
k__Bacteria; p__Proteobacteria; c__Gammaproteobacteria; o__Xanthomonadales; f__Xanthomonadaceae; g__Silanimonas;	62	365	2	24	103	137	4	139
k__Bacteria; p__Proteobacteria; c__Gammaproteobacteria; o__Xanthomonadales; f__Xanthomonadaceae; g__Stenotrophomonas;	301	460	510	480	870	694	303	811
k__Bacteria; p__Proteobacteria; c__Gammaproteobacteria; o__Xanthomonadales; f__Xanthomonadaceae; g__uncultured_bacterium_f_Xanthomonadaceae;	341	445	1313	1753	707	1286	444	228
k__Bacteria; p__Proteobacteria; c__Gammaproteobacteria; o__uncultured_bacterium_c_Gammaproteobacteria; f__uncultured_bacterium_c_Gammaproteobacteria;	220	991	1155	564	1125	161	171	391

Taxonomy	OTUs							
	SM	PA	KD	LU	SP	LV	HE	BR
g__uncultured_bacterium_c_Gammaproteobacteria;								
k__Bacteria; p__Rokubacteria; c__NC10; o__Methylomirabilales; f__Methylomirabilaceae; g__Sh765B-TzT-35;	16	77	0	0	145	16	0	0
k__Bacteria; p__Rokubacteria; c__NC10; o__Rokubacteriales; f__bacterium_WX65; g__uncultured_bacterium_f_bacterium_WX65;	152	3	123	25	55	574	81	7
k__Bacteria; p__Rokubacteria; c__NC10; o__Rokubacteriales; f__uncultured_bacterium_o_Rokubacteriales; g__uncultured_bacterium_o_Rokubacteriales;	1114	682	1271	1520	971	1417	616	204
k__Bacteria; p__Synergistetes; c__Synergistia; o__Synergistales; f__Synergistaceae; g__Aminobacterium;	0	258	1	0	2	0	0	0
k__Bacteria; p__Synergistetes; c__Synergistia; o__Synergistales; f__Synergistaceae; g__Fretibacterium;	50	1	77	0	0	41	1	0
k__Bacteria; p__Synergistetes; c__Synergistia; o__Synergistales; f__Synergistaceae; g__uncultured_bacterium_f_Synergistaceae;	39	56	4	279	1	91	1	0
k__Bacteria; p__Tenericutes; c__Mollicutes; o__Anaeroplasmatales; f__Anaeroplasmataceae; g__Anaeroplasma;	591	3	0	0	1	0	0	3
k__Bacteria; p__Verrucomicrobia; c__Verrucomicrobiae; o__Chthoniobacterales; f__Chthoniobacteraceae; g__Candidatus_Udaeobacter;	16	28	53	51	112	101	33	41
k__Bacteria; p__Verrucomicrobia; c__Verrucomicrobiae; o__Opitutales; f__Opitutaceae; g__Lacunisphaera;	0	1	252	0	2	0	0	0
k__Bacteria; p__Verrucomicrobia; c__Verrucomicrobiae; o__Pedosphaerales; f__Pedosphaeraceae; g__uncultured_bacterium_f_Pedosphaeraceae;	0	0	0	321	2	3	0	0
k__Bacteria; p__Verrucomicrobia; c__Verrucomicrobiae; o__Verrucomicrobiales; f__Akkermansiaceae; g__Akkermansia;	437	2889	464	1462	1560	408	603	773
k__Bacteria; p__WPS-2; c__uncultured_bacterium_p_WPS-2; o__uncultured_bacterium_p_WPS-2; f__uncultured_bacterium_p_WPS-2; g__uncultured_bacterium_p_WPS-2;	119	228	208	167	231	96	325	222
k__Bacteria; p__uncultured_bacterium_k_Bacteria; c__uncultured_bacterium_k_Bacteria; o__uncultured_bacterium_k_Bacteria; f__uncultured_bacterium_k_Bacteria; g__uncultured_bacterium_k_Bacteria;	158	13	384	91	533	950	566	20

*: top 10 bacteria. SM: skeletal muscle, PA: pancreas, KD: kidney, LU: lung, SP: spleen, LV: liver, HE: heart, BR: brain

OTUs: operational taxonomic units.
