# Supplementary material for: The Cellular Microbiome of Visceral Organs: An Inherent Inhabitant of Parenchymal Cells
Source: Microorganisms. 2024 Jun 29;12(7):1333. doi: 10.3390/microorganisms12071333 (PMC11279389; doi:10.3390/microorganisms12071333)
Supplement: Supplementary file 1 [file microorganisms-12-01333-s001.zip › Supplementary Table S2 and Table S3.pdf]

Table S2. Expression of LPS and LTA in visceral organs detected via western blotting and immunofluorescence.

	Brain	Heart	Liver	Spleen	Lung	Kidney	Pancreas	Skeletal Muscle	Stomach	Small Intestine	Large Intestine
**LPS**	+	+	+	+	+	+	+	+	+	+	+
**Location**	Nu.	Nu.	Nu.	Nu./Cy.	Nu./Cy.	Nu.	Nu.	Nu.	Nu.	Nu.	Nu./Cy.
**LTA**	+	+	-	+	+	+	+	+	+	+	+
**Location**	Cy.	Cy.	-	Cy.	Cy.	Cy.	Cy.	Cy.	Cy.	Cy.	Cy.

Nu.: nucleus; Cy.: cytoplasm; “-”: not detected. LPS: lipopolysaccharide, LTA: lipoteichoic acid.

Table S3. Expression of LPS and LTA in cell lines detected via western blotting.

		Hep G2	Huh-7	Hepa 1-6	HSC T6
**Cytoplasm**	LPS	-	+/-	-	-
	LTA	-	-	-	-
**Nucleus**	LPS	+	+	+	+
	LTA	-	-	-	-

“-”: not detected. LPS: lipopolysaccharide, LTA: lipoteichoic acid.
